# Community-based rehabilitation intervention for people with schizophrenia in Ethiopia (RISE): results of a 12-month cluster-randomised controlled trial

**DOI:** 10.1016/S2214-109X(22)00027-4

**Published:** 2022-03-15

**Authors:** Laura Asher, Rahel Birhane, Helen A Weiss, Girmay Medhin, Medhin Selamu, Vikram Patel, Mary De Silva, Charlotte Hanlon, Abebaw Fekadu

**Affiliations:** a Lifespan and Population Health Unit, School of Medicine, University of Nottingham, Nottingham, UK; b WHO Collaborating Centre for Mental Health Research & Capacity Building, Department of Psychiatry, School of Medicine, College of Health Sciences, Addis Ababa University, Addis Ababa, Ethiopia; c Akililu Lemma Institute of Pathobiology, Addis Ababa University, Addis Ababa, Ethiopia; d Centre for Innovative Drug Development and Therapeutic Trials for Africa, Addis Ababa University, Addis Ababa, Ethiopia; e MRC International Statistics and Epidemiology Group, London School of Hygiene and Tropical Medicine, London, UK; f Department of Global Health and Social Medicine, Harvard Medical School, Harvard University, Boston, MA, USA; g Harvard Chan School of Public Health, Harvard University, Boston, MA, USA; h The Wellcome Trust, London, UK; i Centre for Global Mental Health, Department of Health Service and Population Research, Institute of Psychiatry, Psychology and Neuroscience, King's College London, London, UK; j Department of Global Health & Infection, Brighton and Sussex Medical School, Brighton, UK

## Abstract

**Background:**

Community-based rehabilitation (CBR) is recommended to address the social and clinical needs of people with schizophrenia in resource-poor settings. We evaluated the effectiveness of CBR at reducing disability at 12 months in people with schizophrenia who had disabling illness after having had the opportunity to access facility-based care for 6 months

**Methods:**

This cluster-randomised controlled trial was conducted in a rural district of Ethiopia. Eligible clusters were subdistricts in Sodo district that had not participated in the pilot study. Available subdistricts were randomised (in a 1:1 ratio) to either the intervention group (CBR plus facility-based care) or to the control group (facility-based care alone). An optimisation procedure (accounting for the subdistrict mean WHO Disability Assessment Schedule (WHODAS) score and the potential number of participants per subdistrict) was applied for each of the eight health facilities in the district. An independent statistician, masked to the intervention or control label, used a computer programme to randomly choose the allocation sequence from the set of optimal ones. We recruited adults with disabling illness as a result of schizophrenia. The subdistricts were eligible for inclusion if they included participants that met the eligibility criteria. Researchers recruiting and assessing participants were masked to allocation status. Facility-based care was a task-shared model of mental health care integrated within primary care. CBR was delivered by lay workers over a 12-month period, comprising of home visits (psychoeducation, adherence support, family intervention, and crisis management) and community mobilisation. The primary outcome was disability, measured with the proxy-rated 36-item WHODAS score at 12 months. The subdistricts that had primary outcome data available were included in the primary analysis. This study is registered with ClinicalTrials.gov, NCT02160249.

**Findings:**

Enrolment took place between Sept 16, 2015 and Mar 11, 2016. 54 subdistricts were randomised (27 to the CBR plus facility-based care group and 27 to the facility-based care group). After exclusion of subdistricts without eligible participants, we enrolled 79 participants (66% men and 34% women) from 24 subdistricts assigned to CBR plus facility-based care and 87 participants (59% men and 41% women) from 24 subdistricts assigned to facility-based care only. The primary analysis included 149 (90%) participants in 46 subdistricts (73 participants in 22 subdistricts in the CBR plus facility-based care group and 76 participants in 24 subdistricts in the facility-based care group). At 12 months, the mean WHODAS scores were 46·1 (SD 23·3) in the facility-based care group and 40·6 (22·5) in the CBR plus facility-based care group, indicating a favourable intervention effect (adjusted mean difference −8·13 [95% CI −15·85 to −0·40]; p=0·039; effect size 0·35). Four (5%) CBR plus facility-based care group participants and nine (10%) facility-based care group participants had one or more serious adverse events (death, suicide attempt, and hospitalisation).

**Interpretation:**

CBR delivered by lay workers combined with task-shared facility-based care, was effective in reducing disability among people with schizophrenia. The RISE study CBR model is particularly relevant to low-income countries with few mental health specialists.

**Funding:**

Wellcome Trust.

## Introduction

Schizophrenia is a global mental health priority due to the high levels of associated disability, poverty, premature mortality, and human rights abuses.^1^ In low-income and middle-income countries (LMICs), these adverse effects are amplified by the inadequacy and inaccessibility of existing formal care systems. In low-income countries, there are severe shortages of mental health specialists, who are usually concentrated in urban centres. As a result, 89% of people with schizophrenia in low-income countries do not receive evidence-based treatment.^2^ For many people with schizophrenia, functional recovery is made more achievable with psychosocial rehabilitation in addition to pharmacological treatments; yet, psychosocial support is absent in most LMICs.^3^

**Research in context**

**Evidence before this study**

A previous systematic review assessing evidence from randomised controlled trials (RCTs) published before 18 April, 2016, identified 11 RCTs in China, India, Iran, South Africa, and Turkey. Interventions included psychoeducational interventions, multi-component rehabilitation-focused interventions, and case management interventions; and with one exception they were delivered by specialists such as psychiatrists and mental health social workers. The findings were that community-based psychosocial interventions in low-income and middle-income countries (LMICs) are effective in improving symptoms and functioning and reducing hospital admissions in people with schizophrenia. As an update, we searched the Medline and PsychInfo databases from Jan 1, 2016, to March 12, 2021, using the terms “schizophrenia” or “mental disorders” and related terms; and “CBR” or “psychiatric rehabilitation” and related terms; and LMICs, as defined by the World Bank. Two additional RCTs (n=327 and n=60) and one randomised pilot study (n=57) were identified; they were all were set in China. Intervention components included psychoeducation, supportive counselling, family support, social skills training, and vocational skills training as part of the Clubhouse model. Two were delivered in community rehabilitation or health centres and one was delivered through home visits. All three trials found positive intervention effects on symptom severity, readmissions, and functioning. Across the systematic review and updated search, we identified no RCTs conducted in low-income countries, none in which the intervention was delivered as an adjunct to task-shared care, and only one study in which the intervention was delivered by lay workers. Employment assistance, raising community awareness, and signposting to community resources featured in one, two, and five studies respectively; to our knowledge no trial has incorporated active mobilisation of community members or organisations to support individuals with schizophrenia.

**Added value of this study**

To our knowledge, the RISE trial is the first RCT to evaluate the effectiveness of any type of psychosocial intervention for people with schizophrenia in a low-income country, and it is the first to assess the effectiveness of comprehensive community-based rehabilitation (CBR) for schizophrenia, including a structured community mobilisation component, in any setting globally. We found that CBR delivered by lay workers combined with facility-based care delivered predominantly in primary care was effective in reducing disability (particularly participation and social interactions), symptom severity, and caregiver tension and worrying; and effective in increasing antipsychotic medication adherence and attendance to facility-based care for mental health among people with schizophrenia at 12 months follow-up. The use of lay workers and non-specialist supervisors to deliver CBR enhances the minimal evidence on task-shared approaches to the care of people with schizophrenia and demonstrates that psychosocial support can be successfully delivered in resource-poor settings.

**Implications of all the available evidence**

In LMICs, community-based psychosocial interventions should be provided as an adjunct to facility-based treatment services providing access to antipsychotic medication. Combining psychosocial and pharmacological support is likely to produce the best treatment outcomes in terms of functional recovery and symptom severity. The intervention content and delivery personnel can be tailored to the resources available and the social context. Where there are few mental health specialists, lay workers can successfully provide psychosocial support as an adjunct to task-shared facility-based care.

Community-based rehabilitation (CBR) is a rights-based approach that is successfully used to support the rehabilitation and social inclusion of people with disabilities in LMICs.^4^ CBR champions a social model of disability, which states that disability is a product of both the environment and illness. It therefore aims to reduce disability through shaping the social world, using community mobilisation, and individual and family support. Alongside facility-based care and antipsychotic medication, delivered predominantly in primary care**,** CBR is recommended by WHO as a suitable psychosocial intervention for people with schizophrenia in LMICs.^5^ As CBR can be delivered by lay workers,^4^ it offers an affordable and accessible means to address the complex social, economic, and clinical needs of people with schizophrenia in resource-poor settings.

Community-based psychosocial interventions positively affect symptom severity, functioning, and hospital admissions among people with schizophrenia in LMICs.^3^ Research to date has two major limitations. First, all randomised trials to date were set in middle-income countries as an adjunct to well-resourced and accessible mental health care such as free antipsychotic medication and access to psychiatrists; and with one exception,^6^ none were delivered by lay workers. Second, with some exceptions,^7^, ^8^ there has been little evaluation of the community mobilisation aspects of CBR or similar models.^3^

To address this evidence gap, we developed the Rehabilitation Intervention for People with Schizophrenia in Ethiopia (RISE) CBR intervention over an 18-month formative phase,^9^ then demonstrated its acceptability and feasibility in a 12-month pilot study.^10^ The RISE intervention was designed to meet the needs of people with schizophrenia in a rural Ethiopian district, a low-income setting with poor mental health care infrastructure and high poverty levels, which can impede care engagement and recovery. Unlike previous evaluations, CBR was devised as an adjunct to task-shared facility-based care for schizophrenia, integrated within primary health-care centres, and was not supervised by mental health specialists. This facility-based model was implemented as part of the Programme for Improving Mental Health Care (PRIME).^11^ A distinctive component of the RISE intervention is its aim to shape individuals’ social environment by enhancing community support and reducing stigmatising attitudes.^4^

The aim of this trial was to evaluate the effectiveness of CBR plus facility-based care compared with facility-based care alone at reducing disability at 12 months in people with schizophrenia. We focused on individuals who did not respond to or engage with treatment, to reflect the target group for CBR if it were scaled up in a resource-poor setting.

## Methods

### Study design and participants

We conducted a cluster-randomised controlled trial in Sodo district, Gurage Zone, Southern Nations, Nationalities, and Peoples’ Region in Ethiopia. Sodo district has a total population of 170 000 people in 58 subdistricts. It is a socioeconomically deprived area with a largely agrarian economy, approximately 55% literacy, and several remote rural areas. Primary care is delivered by nurses and health officers at seven health centres and one primary hospital. Care costs are usually out-of-pocket with a fee waiver available for the poorest. The study protocol was previously published^12^ (appendix pp 1–2 for amendments). Ethics approval was obtained from Institutional Review Boards at the London School of Hygiene & Tropical Medicine, Addis Ababa University College of Health Sciences, and the Ethiopian National Research Ethics Review Committee.

Randomisation units were subdistricts of Sodo district. We identified 54 available subdistricts after subtracting the four pilot subdistricts.^10^ Subdistricts were excluded at screening and enrolment stages if no eligible participants were identified (figure). People with suspected schizophrenia in Sodo district were identified by PRIME using key community informants and health-extension workers who had received half a day of training in the typical presentations of schizophrenia (ie, the key informant method). This information was added to a database. Trained psychiatric nurses in the PRIME study used the Operational Criteria for Research (OPCRIT) diagnostic interview (which applies Diagnostic and Statistical Manual of Mental Disorders fourth edition [DSM-IV] criteria),^13^ and those with a confirmatory diagnosis of schizophrenia were recruited to the PRIME study. Facility-based care was available for people in the PRIME study with a confirmed diagnosis of schizophrenia. The PRIME study started 6 months before RISE trial recruitment. RISE study participants had therefore had the opportunity to access facility-based care for 6 months before trial recruitment. There was some overlap between the studies as the PRIME study duration was 12 months.

**Figure:**
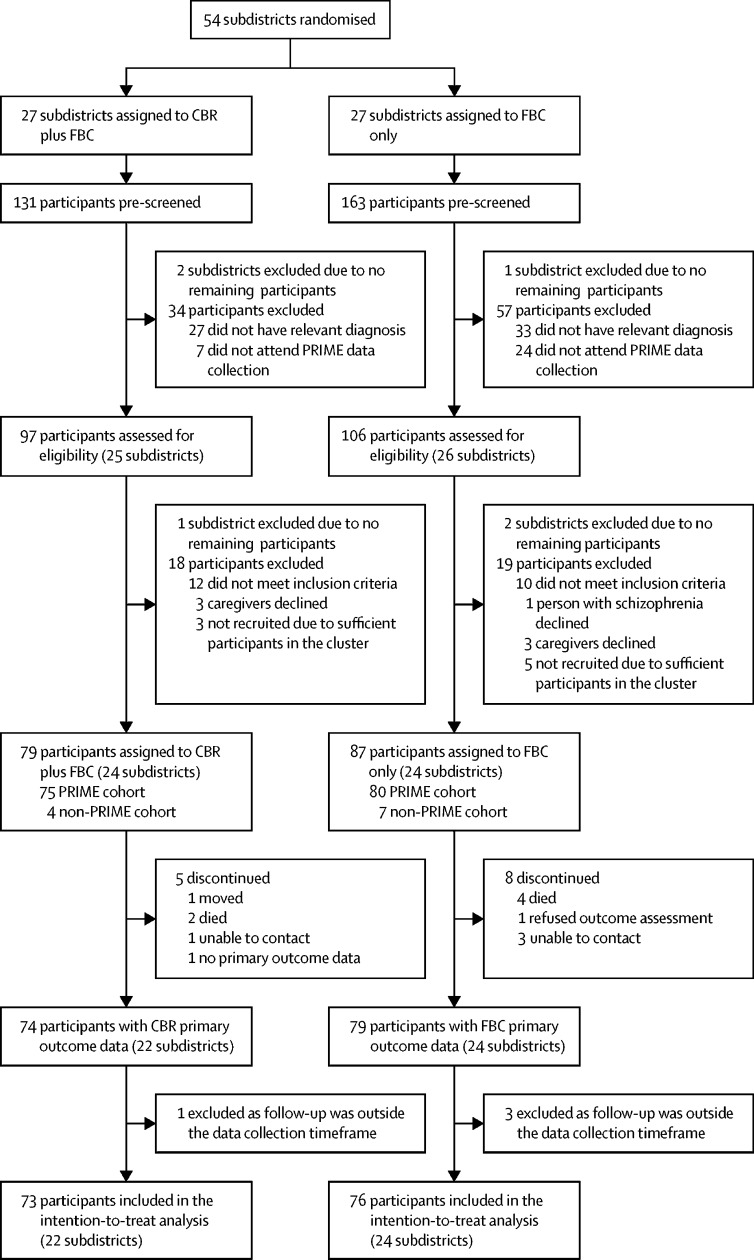
Trial profile CBR=community-based rehabilitation. FBC=facility-based care.

RISE study participants included individuals who were participating in the PRIME study and individuals who had not participated in the PRIME study but had been identified by PRIME as having suspected schizophrenia (these participants had not attended facility-based care). Participants from the PRIME study were recruited at the PRIME 6-month data collection (or on a separate occasion if they did not attend) and individuals who were not participants in the PRIME study were recruited using the PRIME database of people with suspected schizophrenia. The initial contact for these patients was made by phone call or home visit by the data collector, and a full eligibility assessment visit was subsequently arranged.

For each participant, one primary caregiver was identified who met the criteria of being aged 18 years or older and providing regular support (eg, sustenance). Caregivers could be a spouse, relative, or friend. Potential participants were requested to attend data collection with a caregiver, who was screened for these criteria. If the original caregiver was unavailable at the 12-month data collection, caregiver-reported data were collected from a different caregiver meeting the criteria.

Study participants with a schizophrenia diagnosis who were recruited during participation in the PRIME study underwent full RISE study eligibility assessment by the RISE trial nurse using data gathered at 6 months during the PRIME study. Study participants who were not from the PRIME study were assessed for eligibility, which included the diagnostic OPCRIT interview.

Participants were eligible to enrol in the study if they were aged 18 years or older; were a participant of the PRIME cohort study or residing in Sodo district but not engaged in facility-based care; had a diagnosis of schizophrenia, schizoaffective disorder, or schizophreniform disorder; had no intention to leave the subdistrict; had a primary caregiver willing to participate; and had one or more markers of severe, disabling, or enduring illness (Brief Psychiatric Rating Scale-Expanded [BPRSE] score ≥52, proxy or self-rated 36-item WHO Disability Assessment Schedule [WHODAS] 2.0 score ≥35, continuous illness lasting 6 months, symptomatic for ≥3 of last 6 months, or Clinical Global Impression [CGI] severity score ≥3).

Written informed consent was sought from each eligible participant and caregiver by a trial nurse. For those without capacity to consent, permission was sought from the caregiver and assent from the person with schizophrenia. Individuals were not recruited if they expressed unwillingness to participate. If the participant was unable to write, a thumb impression and a witness's signature were recorded.

### Randomisation and masking

Randomisation of subdistricts was carried out before participant recruitment by an independent statistician. 54 subdistricts were randomised (in a 1:1 ratio) to either the intervention (CBR plus facility-based care group) or to the control group (facility-based care alone group). Cluster randomisation was chosen because CBR includes community-level elements.

To prevent an imbalance for potential confounding factors an independent statistician employed an optimisation algorithm calculated for subdistrict mean WHODAS score at PRIME baseline and the potential number of participants per subdistrict. Although not all patients had a PRIME baseline WHODAS score, the majority of participants did; therefore, we deemed that it was appropriate to use these scores to ensure balance between the treatment groups.

They applied the procedure for each of the eight health facilities and used a computer program to randomly choose the allocation sequence from the set of optimal ones. The statistician was masked to the intervention or control label. Researchers responsible for recruiting participants and collecting outcome data were masked to allocation status. To minimise unmasking, participants and CBR workers were requested not to divulge allocation status to researchers, data collectors were assigned to subdistricts where they had no personal links, and primary outcome data were collected first at each assessment. All coauthors (except RB) remained masked until the final analysis was complete.

### Procedures

Facility-based care is a stepped care model. The majority of care was delivered in primary care, which comprised prescription of antipsychotic medication, and psychoeducation by nurses and health officers trained for 2 weeks in the WHO mental health Gap Action Programme-Intervention Guide (mhGAP) supervised by a psychiatric nurse. The frequency of contact was determined by clinical need, and there was no minimum requirement. Primary care staff could refer participants to psychiatric nurse-led outpatient care (secondary care) or psychiatrist-led inpatient care (tertiary care). Health extension workers in each subdistrict were trained in raising awareness of the causes and treatment of mental health disorders. A district-level community-advisory board oversaw implementation of the facility-based care model.

CBR was delivered by 11 CBR workers, each covering a catchment area of one, two, or three subdistricts linked to a health centre. CBR delivery lasted 12 months, commencing immediately after trial recruitment. CBR workers supported a median of seven participants (range 4–11). CBR workers were lay people recruited from the local area with at least 10 years of education but no previous mental health care experience. They received 5 weeks initial manualised training (approximately 150 h) in CBR delivery, including basic counselling and problem-solving techniques, followed by monthly half-day additional training.^14^ Training was delivered by psychiatrists and CBR coordinators and was split between classroom teaching and fieldwork. In the pre-trial pilot study CBR workers delivered CBR to one family for 6 months.^10^

CBR visits took place at the participants’ home and lasted 30–90 mins. The intervention was recovery-oriented, rights-based and emphasised social inclusion. Topics included psychoeducation, adherence support, family intervention, crisis management, support returning to work and social activities, and dealing with stigma and stress. In phase 1 (2–3 months duration), home visits were every 1–2 weeks, and the focus was on engagement and addressing core needs. In phase 2 (5–6 months duration), home visits were every 2 weeks. A subset from 11 optional modules were selected to address individual goals. In phase 3 (approximately 4 months duration), the emphasis was on maintaining progress. CBR workers met with community members to mobilise resources for individual participants—eg, treatment costs, food, social support, or family mediation. CBR workers supported facility-based care engagement—eg, attempting to obtain medication fee waivers. At a subdistrict level, CBR workers conducted community mobilisation (eg, meetings raising public awareness and engaging with community leaders) and ran family support groups. Two supervisors, who were not mental health specialists, oversaw the frequency and content of home visits. In addition to regular appointments, CBR workers referred participants to the health centre if suicidal intent, relapse, or medication side-effects were identified.

Process data were recorded at the levels of participant (home visits and community mobilisation), subdistrict (community mobilisation and family group meetings), and CBR worker (Enhancing Assessment of Common Therapeutic Factors-Ethiopia [ENACTE] competency assessments,^14^ conducted by independent psychiatric nurses). Minimum adequate CBR participation was predefined as ten or more home visits and optimal CBR participation was predefined as 21 or more home visits.

Data were collected at baseline, 6 months, and 12 months (primary endpoint) at the participant's home or health centre. Sociodemographic information was collected at the PRIME cohort study baseline, or (for participants not in the PRIME cohort study) at RISE trial baseline. Self-rated WHODAS data was collected at PRIME baseline and used for the optimisation algorithm employed in randomisation. Self-reported prescription of antipsychotic medication (all timepoints) and receipt of free medication (12 months only) was recorded.

### Outcomes

All outcomes were measured at an individual level. The primary outcome was disability, measured with the proxy-rated 36-item WHODAS 2.0 total score at 12 months (with a prespecified timeframe of 10 weeks either side of the 12 months), calculated using item-response theory-based scoring. Normative data indicate 95% of the general population score less than 50 (range 0–100).^15^ Domain scores for understanding and communication, getting around, self-care, getting along with people, life activities (household and work), and participation in society were secondary outcomes. WHODAS 2.0 is an instrument for assessing disability relating to any health condition across cultures. Sociocultural adaptation and validation of the WHODAS in people with schizophrenia has been completed in Ethiopia.^16^ The rationale for using the proxy version, completed by a caregiver, was that reporting can vary among people with schizophrenia according to their mental state, and the proxy version showed a greater responsiveness to change in a local study.^16^

Secondary outcomes were 6-month proxy-rated WHODAS total and domain scores; 6 and 12-month symptom severity (CGI);^17^ and 12-month self-rated WHODAS total and domain scores, proxy and self-rated days unable to work in the past month, symptom severity (BPRSE),^18^ Butajira Functioning Scale (BFS) score (a locally developed scale),^19^ relapse (Life Chart Schedule),^20^ health facility attendance for mental health (adapted Client Service Receipt Inventory [CSRI]), medication non-adherence (ordinal and 4-item Morisky Medication Adherence scales), physical restraint, discrimination (Discrimination and Stigma Scale-12),^21^ and employment. Caregiver outcomes were caregiving burden (Involvement Evaluation Questionnaire [IEQ]) and mood (Patient Health Questionnaire-9 [PHQ-9]),^22^ shown in the appendix (pp 2–3). We systematically assessed for the serious adverse events: death (by suicide or any cause), suicide attempts, and hospital admissions (due to suicide attempt, serious side effect of anti-psychotic medication, or any other serious medical emergency). We also recorded any other adverse events that were detected. Lay data collectors collected all data except for symptom severity, relapse, and prescription of medication, which were collected by psychiatric nurses.

### Statistical analysis

Assuming a 23% attrition rate, we aimed to recruit 182 participants to provide a sample size for analysis of 140 participants with schizophrenia in 54 subdistricts. This sample size provides 85% power to detect a 20% absolute difference in WHODAS scores between the treatment groups, with 5% significance, assuming a mean WHODAS score of 50 in the control (facility-based care) group, a co-efficient of variation of 0·14, and a within-cluster SD of 16.

Initial analyses compared the baseline characteristics of participants and subdistricts with the completion of 12-month outcome assessments and between treatment groups. Analyses were done in participants according to the group that they were randomised to. Outcome measures were summarised at baseline, 6-month, and 12-month data points by treatment group. For continuous outcomes, we estimated intervention effects using linear mixed-effects regression and reported them as minimally-adjusted mean differences, fully-adjusted mean differences (FAMDs), and effect sizes defined as standardised mean differences, with 95% CIs. For binary outcomes, we reported intervention effects as minimally-adjusted, fully-adjusted odds ratios (FAORs), and 95% CIs estimated from logistic random effect regression models. Minimally-adjusted models included baseline proxy-rated WHODAS total score, health centre (fixed effect), and subdistrict (random effect). Fully adjusted models included variables associated with missingness (threshold p<0·10), and variables deemed unbalanced between groups at baseline.

We assessed modification of treatment effect by predefined effect modifiers (sex and baseline symptom severity, disability, alcohol use disorder, social support, and socioeconomic status) using the likelihood ratio test. If two or less WHODAS items were missing, the mean score across all domain items was assigned to the missing items.

Process data were summarised for participant, subdistrict, and CBR worker levels. We calculated the proportion of participants prescribed antipsychotic medication at each timepoint and accessing free medication at 12 months; the numbers and proportions of participants lost to follow-up, and the reasons overall and by group; the proportion of participants for whom the assessor was unmasked; and the numbers and proportions of participants with each serious adverse event type and any serious adverse event by group.

The primary analyses were complete case (ie, we only included participants with data on the variables of interest). Sensitivity analyses included inclusion of participants with data collected within the timeframe of 6 weeks either side of the 12 months or at any time; exclusion of caregivers who differed between baseline and 12 months; and multiple imputation of missing data with a linear or logistic imputation model as appropriate, adjusting for factors associated with missingness, for the imputation with 50 imputed datasets. Statistical analyses were done with Stata, version 15. All analyses were prespecified, except the description of medication prescription. An independent Data Safety and Monitoring Board oversaw the study. The trial is registered with ClinicalTrials.gov, NCT02160249.

### Role of the funding source

The funder of the study had no role in study design, data collection, data analysis, data interpretation, or writing of the report.

## Results

Participants were enrolled between Sept 16, 2015, and March 11, 2016, (figure). Of the 54 available subdistricts, 27 were randomly assigned to the CBR plus facility-based care group and 27 to the facility-based care group. A total of 294 potential participants were prescreened and 91 were excluded. A further 37 individuals were not enrolled; of these, one participant and six (3%) caregivers of 203 participants and linked caregivers declined (three caregivers in the CBR plus facility-based care group and one participant and three caregivers in the facility-based care group). Three subdistricts were excluded at each of the prescreening and enrolment stages because there were no eligible participants. Therefore, 48 of 54 potential subdistricts were included. 24 subdistricts (79 participants) were assigned to the CBR plus facility-based care group and 24 subdistricts (87 participants) were assigned to the facility-based care group. Primary outcome data collected in the predefined timeframe were available for 73 (92%) of 79 participants in the CBR plus facility-based care group (22 subdistricts) and 76 (87%) of 87 participants in the facility-based care group (24 subdistricts); see figure).

Participants had a median age of 31·4 years (range 18·0–80·0). At baseline there were high levels of disability (mean proxy-rated WHODAS 51·5 [SD 23·6]), and 132 (80%) of 166 participants reported continuous illness over the previous 6 months. Participants in the facility-based care group were more likely than those in the CBR plus facility-based care group to be female, to have lower household socio-economic status, to be unemployed, and to have social support. Caregivers in the facility-based care group were less likely to be unemployed or be depressed than caregivers in the CBR plus facility-based care group (table 1). At baseline, 38 (44%) of 87 participants in the facility-based care group and 42 (54%) of 79 participants in the CBR plus facility-based care group had been prescribed antipsychotic medication, increasing to 39 (49%) of 79 participants in the facility-based care group and 58 (77%) of 75 participants in the CBR plus facility-based care group at 12-months (appendix p 3). Almost all prescriptions were for oral first-generation antipsychotics (chlorpromazine and haloperidol). At 12-months, free antipsychotic medication was available to 24 (30%) of 79 participants in the facility-based care group and 37 (50%) of 74 participants in the CBR plus facility-based care group (appendix p 3).

**Table 1:** Baseline characteristics of RISE trial participants by treatment group

		**Facility-based care group (n=87)**	**CBR plus facility-based care group (n=79)**
**Participant**			
Sex			
	Male	51 (59%)	52 (66%)
	Female	36 (41%)	27 (34%)
Age, years (median [IQR])		33 (25–40)	30 (25–45)
Marital status*			
	Single	41/80 (51%)	38/75 (51%)
	Has a partner (married or married but not living together)	26/80 (33%)	25/75 (33%)
	Separated, divorced, or widowed	13/80 (16%)	12/75 (16%)
Occupation			
	No occupation	15 (17%)	9 (11%)
	Home worker	33 (38%)	27 (34%)
	Unskilled labourer	34 (39%)	42 (53%)
	Other	5 (6%)	1 (1%)
Education status*			
	No formal education	46/80 (58%)	36/75 (48%)
	Primary education	22/80 (28%)	35/75 (47%)
	Secondary education and above	12/80 (15%)	4/75 (5%)
Socioeconomic status*			
	Higher (poverty index ≤3)	42/80 (53%)	47/74 (64%)
	Lower (poverty index >3)	38/80 (48%)	27/74 (36%)
Residence*			
	Urban	11/80 (14%)	8/74 (11%)
	Rural	69/80 (86%)	66/74 (89%)
Travel time to nearest health facility*			
	≤60 mins	51/80 (64%)	48/75 (64%)
	61–120 mins	13/80 (16%)	17/75 (23%)
	≥121 mins	16/80 (20%)	10/75 (13%)
Diagnosis			
	Schizophrenia	70 (80%)	68 (86%)
	Schizoaffective or schizophreniform disorder	17 (20%)	11 (14%)
Duration of illness in years* (median [IQR])		4 (1·9–9); n=64	3·7 (1·5–7·3); n=55
Comorbid medical disorder*			
	No	69/76 (91%)	69/72 (96%)
	Yes	7/76 (9%)	3/72 (4%)
Proxy-rated total WHODAS (mean [SD])		52·6 (23·6)	50·2 (23·6)
BPRSE total (mean [SD])		47·2 (13·4); n=85	48·9 (14·2); n=75
CGI			
	Normal or borderline score	6 (7%)	2 (3%)
	At least mildly ill (score ≥3)	81 (93%)	77 (97%)
Illness course lasting 6 months (LCS)			
	Episodic	3 (3%)	4 (5%)
	Continuous	66 (76%)	66 (84%)
	Never psychotic	18 (21%)	9 (11%)
Antipsychotic medication adherence			
	All or most of the time	35/83 (42%)	36/77 (47%)
	Sometimes, occasionally, or not at all	48/83 (58%)	41/77 (53%)
Engagement with care			
	No healthcare attendance and no medication adherence	32/83 (39%)	30/77 (39%)
	Either healthcare attendance or medication adherence	23/83 (28%)	17/77 (22%)
	Healthcare attendance and medication adherence	28/83 (34%)	30/77 (39%)
AUDIT total ≥8			
	No	70/86 (81%)	58/74 (78%)
	Yes	16/86 (19%)	16/74 (22%)
Restrained last 6 months			
	No	82 (94%)	75 (95%)
	Yes	5 (6%)	4 (5%)
Any experience of discrimination last 6 months			
	No	40 (46%)	38 (48%)
	Yes	47 (54%)	41 (52%)
Unemployed			
	No	32 (37%)	38 (48%)
	Yes	55 (63%)	41 (52%)
Social support			
	Poor	23 (26%)	35 (44%)
	Intermediate	49 (56%)	31 (39%)
	Strong	15 (17%)	13 (17%)
**Caregiver**			
Mean total IEQ score (mean [SD])		40·1 (16·0)	40·7 (19·4)
PHQ-9 score ≥5			
	No	57 (66%)	36 (46%)
	Yes	30 (34%)	43 (54%)
Unemployed			
	No	55 (63%)	43 (54%)
	Yes	32 (37%)	36 (46%)
**Sub-district**			
Location			
	Urban	1/24 (4%)	2/24 (8%)
	Rural	23/24 (96%)	22/24 (92%)
Baseline number of participants (median [IQR])		3 (1·5–4·5)	2·5 (1–5)
Proxy-rated total WHODAS (median [IQR])		52·8 (45·2–61·1)	54·1 (41·4–62·4)

Data are n or n/N (%) unless otherwise indicated. Percentage totals might not equal 100% due to rounding. CBR=community-based rehabilitation. WHODAS=World Health Organization Disability Assessment Schedule. BPRSE=Brief Psychiatric Rating Scale Expanded. CGI=Clinical Global Impression scale. LCS=Life Chart Schedule. AUDIT=Alcohol Use Disorder Identification Test. IEQ=Involvement Evaluation Questionnaire. PHQ-9=Patient Health Questionairre-9.

* Data collected at PRIME cohort study baseline.

There was evidence of a favourable intervention effect on the primary outcome, proxy-rated WHODAS score at 12 months. The mean WHODAS scores at 12 months were 46·1 (SD 23·3) in the facility-based care group and 40·6 (22·5) in the CBR plus facility-based care group (FAMD −8·13 [95% CI −15·85 to −0·40]; p=0·039; effect size=0·35; table 2). There were also favourable effects on the secondary outcomes of proxy-rated WHODAS domain scores of cognition (FAMD −9·65 [95% CI −19·31 to 0·01]; p=0·050), getting along (FAMD −14·32 [–26·60 to −2·05]; p=0·022), participation (FAMD −8·86 [–16·81 to −0·91]; p=0·029), and illness severity measured with the CGI (FAOR 0·26 [95% CI 0·09–0·81]; p=0·019). The FAMD on the BPRSE score was −5·31 (95% CI −10·86 to 0·23; p=0·060). There was no evidence of intervention effects on discrimination (FAOR 2·26 [95% CI 0·88–5·81]; p=0·089), restraint (FAOR 2·72 [0·34–21·83]; p=0·35), or the WHODAS domains of work (FAMD −9·22 [95% CI −20·00 to 1·57]; p=0·094), household (FAMD −7·37 [–18·72 to 3·98]; p=0·20) and self-care (FAMD −3·44 [–14·4 to 7·56]; p=0·54) at 12 months (table 2); or WHODAS total score or illness severity at 6 months (appendix p 4). Participants in the CBR plus facility-based care group were less likely to have no attendance to a health facility for mental health in the past 3 months than participants in the facility-based care group (FAOR 0·19 [95% CI 0·07–0·54]; p=0·0020), and they were less likely to report poor frequency of adherence to antipsychotic medication (FAOR 0·19 [95% CI 0·07–0·51]; p=0·0010). However, no effect was observed on adherence behaviours measured with the Morisky Scale (FAOR 0·63 [95% CI 0·25–1·56]; p=0·32). Participants in the CBR plus facility-based care group were more likely to be unemployed compared to participants in the facility-based care group (FAOR 3·80 [95% CI 1·10–13·07; p=0·034). There were beneficial intervention effects on caregiver burden in the IEQ domains of tension (FAMD −1·83 [95% CI −3·62 to −0·05]; p=0·044) and worrying (FAMD −2·17 [–4·26 to −0·08]; p=0·042; table 3).

**Table 2:** Primary and secondary outcomes in people with schizophrenia at 12 months (plus or minus 10 weeks)

		**Facility-based care (n=76)**	**CBR plus facility-based care (n=73)**	**Minimally adjusted analysis**		**Fully adjusted analysis**		**Effect size (95% CI)**
				Mean difference or odds ratio (95% CI)*	p value	Mean difference or odds ratio (95% CI)†	p value	
**Primary outcome**								
Proxy-rated WHODAS-36 total score		46·1 (23·3)	40·6 (22·5)	−6·23 (−13·82 to 1·35)	0·11	−8·13 (−15·85 to −0·40)	0·039	0·35 (0·02 to 0·67)
**Secondary outcomes**								
Proxy-rated WHODAS-36 domain scores								
	Cognition	53·9 (29·9)	47·1 (30·3)	−5·97 (−14·75 to 2·81)	0·18	−9·65 (19·31 to 0·01)	0·050	0·32 (−0·01 to 0·64)
	Mobility	21·5 (24·4)	19·4 (21·3)	−2·76 (−12·02 to 6·49)	0·56	−3·07 (−13·52 to 7·38)	0·57	0·13 (−0·19 to 0·46)
	Self care	32·8 (29·0)	30·8 (24·3)	−2·48 (−13·34 to 8·38)	0·65	−3·44 (−14·4 to 7·56)	0·54	0·13 (−0·19 to 0·45)
	Getting along	49·3 (31·1)	38·8 (30·9)	−12·20 (−22·38 to −2·03)	0·019	−14·32 (−26·60 to −2·05)	0·022	0·45 (0·12–0·77)
	Life activities: household	66·6 (33·4)	61·1 (34·8)	−6·09 (−16·85 to 4·67)	0·27	−7·37 (−18·72 to 3·98)	0·20	0·22 (−0·11 to 0·54)
	Life activities: work	62·7 (32·0)	56·4 (32·6)	−6·51 (−16·49 to 3·47)	0·20	−9·22 (−20·00 to 1·57)	0·094	0·28 (−0·04 to 0·60)
	Participation	41·7 (23·1)	36·5 (24·5)	−6·73 (−14·7 to 1·19)	0·096	−8·86 (−16·81 to −0·91)	0·029	0·36 (0·04 to 0·69)
Proxy-rated number of days unable to work last month		8 (2·5 to 20)	7 (3–15)	−2·64 (−6·22 to 0·95)	0·15	−3·04 (−7·10 to 1·03)	0·14	0·30 (−0·03 to 0·62)
Self-rated WHODAS total score		34·2 (24·2); n=57	29·8 (19·8); n= 59	−3·65 (−11·84 to 4·54)	0·38	−4·77 (−13·81 to 4·26)	0·30	0·21 (−0·15 to 0·58)
Self-rated WHODAS domain scores								
	Cognition	35·3 (29·9); n=57	32·8 (26·9); n=59	0·83 (−10·11 to 11·76)	0·88	−2·07 (−14·15 to 10·00)	0·74	0·07 (−0·29 to 0·44)
	Mobility	15·0 (21·3); n=57	17·8 (21·6); n=59	0·59 (−9·30 to 10·48)	0·91	2·53 (−7·39 to 12·45)	0·62	0·12 (−0·25 to 0·48)
	Self-care	21·2 (24·1); n=57	19·5 (18·3); n=59	−0·83 (−10·26 to 8·60)	0·86	−1·16 (−10·22 to 7·90)	0·80	0·05 (−0·31 to 0·42)
	Getting along	34·4 (32·0); n=57	25·0 (24·5); n=59	−6·82 (−17·59 to 3·95)	0·22	−7·76 (−19·60 to 4·08)	0·20	0·27 (−0·10 to 0·63)
	Life activities: household	55·6 (36·4); n=57	47·5 (34·4); n=59	−7·19 (−19·85 to 5·47)	0·27	−11·96 (−25·92 to 1·99)	0·093	0·33 (−0·03 to 0·70)
	Life activities: work	50·3 (33·5); n=57	42·7 (33·0); n=59	−7·24 (−18·95 to 4·47)	0·23	−10·05 (−22·95 to 2·85)	0·13	0·30 (−0·07 to 0·66)
	Participation	33·0 (23·7); n=57	27·0 (21·2); n=59	−5·84 (−14·33 to 2·64)	0·18	−5·70 (−15·82 to 4·41)	0·27	0·25 (−0·11 to 0·62)
Self-rated number of days unable work last month		4 (0 to 10); n=57	5 (0 to 10); n=59	−1·92 (−5·19 to 1·35)	0·25	−2·06 (−5·35 to 1·23)	0·22	0·27 (−0·10 to 0·63)
Proxy-rated Butajira Functioning Scale		98·5 (38·7)	88·4 (35·8)	−7·15 (−20·11 to 5·81)	0·28	−5·39 (−18·96 to 8·18)	0·44	0·14 (−0·18 to 0·46)
Symptom severity								
	BPRSE score‡	45·4 (13·7); n=68	41·6 (15·6); n=67	−4·81 (−10·08 to 0·47)	0·074	−5·31 (−10·86 to 0·23)	0·060	0·36 (−0·01 to 0·70)
	At least mildly ill (CGI score ≥3)‡	63 (83%)	48 (66%)	OR 0·37 (0·16 to 0·85)	0·020	OR 0·26 (0·09 to 0·81)§	0·019	..
Relapse								
	Relapsed	15 (21%); n=73	17 (25%); n=67	OR 1·06 (0·38 to 2·97)	0·91	OR 1·46 (0·46 to 4·64)¶	0·52	..
Medication & health service use								
	Non-adherent (takes medication sometimes, occasionally, or never)	43 (57%)	21 (29%)	OR 0·16 (0·05 to 0·51)	0·0020	OR 0·19 (0·07 to 0·51)	0·0010	..
	Any non-adherent behaviour	46 (61%)	34 (47%)	OR 0·50 (0·24 to 1·07)	0·08	OR 0·63 (0·25 to 1·56)	0·32	..
	No attendance at a health facility for mental health in the last 3 months	36 (47%)	15 (21%)	OR 0·23 (0·10 to 0·54)	0·0010	OR 0·19 (0·07 to 0·54)	0·0020	..
Physical restraint								
	Restrained in the last 6 months	5 (7%)	5 (7%)	OR 1·09 (0·23 to 5·15)	0·91	OR 2·72 (0·34 to 21·83)¶	0·35	..
Discrimination								
	Any experience of discrimination in the last 6 months	39 (51%)	46 (63%)	OR 2·08 (0·98 to 4·42)	0·057	OR 2·26 (0·88 to 5·81)	0·089	..
Economic activity								
	Unemployed	47 (62%)	51 (70%)	OR 1·76 (0·69 to 4·49)	0·24	OR 3·80 (1·10 to 13·07)	0·034	..

Data are n (%), mean (SD), or median (IQR) unless otherwise indicated. Continuous outcomes display adjusted mean differences and binary outcomes display odds ratio. CBR=community-based rehabilitation. WHODAS= World Health Organization Disability Assessment Schedule. BPRSE=Brief Psychiatric Rating Scale Expanded. CGI=Clinical Global Impression scale. IEQ=Involvement Evaluation Questionnaire. PHQ-9=Patient Health Questionnaire-9.

* Adjusted for sub-district (cluster) as random effect and health centre and baseline score of outcome as fixed effects.

† Unless otherwise stated, adjusted for sub-district (cluster) as a random effect and health centre, baseline score of outcome, baseline disability (proxy-rated total WHODAS), sex, age, residence, baseline socio-economic status, baseline illness course, baseline caregiver burden (IEQ), illness duration, baseline employment status, baseline caregiver employment status, baseline social support, and baseline caregiver depression (PHQ-9) as fixed effects. Illness course and social support reduced to two categories to avoid problems with data sparsity.

‡ For BPRSE and CGI (clinician administered interview) one participant differs to all other outcomes (lay data collector interview).

§ Fully adjusted model excludes residence due to data sparsity.

¶ Fully adjusted model excludes socioeconomic status due to data sparsity.

**Table 3:** Secondary outcomes in caregivers at 12 months (plus or minus 10 weeks)

	**Facility-based care (n=76)**	**CBR plus facility-based care (n=73)**	**Minimally adjusted analysis**		**Fully adjusted analysis**		**Effect size (95% CI)**
			Mean difference or odds ratio (95% CI)*	p value	Mean difference or odds ratio (95% CI)†	p value	
**Caregiver depression**							
PHQ-9 score	4·9 (3·3)	4·8 (3·2)	−0·66 (−2·05 to 0·73)	0·35	−0·52 (−1·72 to 0·69)	0·40	0·16 (−0·16 to 0·48)
PHQ-9 score ≥ 5	35 (46%)	38 (52%)	OR 0·88 (0·34 to 2·28)	0·79	OR 0·94 (0·32 to 2·81)	0·91	..
**Caregiver caring burden**							
IEQ urging domain	12·1 (6·1)	14·1 (6·4)	2·23 (−0·33 to 4·79)	0·088	1·44 (−0·88 to 3·77)	0·22	0·23 (−0·55 to 0·09)
IEQ supervision domain	6·4 (5·2)	7·2 (5·3)	0·92 (−0·92 to 2·76)	0·33	0·29 (−1·74 to 2·33)	0·78	0·06 (−0·38 to 0·27)
IEQ tension domain	6·6 (5·1)	5·6 (5·5)	−1·59 (−3·20 to 0·02)	0·053	−1·83 (−3·62 to −0·05)	0·044	0·34 (0·02 to 0·66)
IEQ worrying domain	10·3 (6·2)	9·5 (6·6)	−1·18 (−3·51 to 1·15)	0·32	−2·17 (−4·26 to −0·08)	0·042	0·34 (0·01 to 0·66)
Reduced work due to caring	25 (33%)	23 (32%)	OR 0·94 (0·39 to 2·23)	0·88	OR 0·92 (0·35 to 2·44)	0·87	..

Data are n (%) or mean (SD) unless otherwise indicated. Continuous outcomes display adjusted mean differences and binary outcomes display odds ratio. PHQ-9=Patient Health Questionairre-9. IEQ=Involvement Education Questionnaire.

* Adjusted for subdistrict (cluster) as random effect and health centre and baseline score of outcome as fixed effects.

† Adjusted for subdistrict (cluster) as a random effect and health centre, baseline score of outcome, baseline disability (proxy-rated total WHODAS), sex, age, residence, baseline socio-economic status, baseline illness course, baseline caregiver burden (IEQ), illness duration, baseline employment status, baseline caregiver employment status, baseline social support, and baseline caregiver depression (PHQ-9) as fixed effects. Illness course and social support reduced to two categories to avoid problems with data sparsity.

In the CBR plus facility-based care group, 71 (90%) of 79 participants reached minimum adequate CBR participation (≥10 visits), whereas 40 (51%) of 79 participants reached optimal CBR participation (≥21 visits). One or more meetings to mobilise specific community resources was held for 45 (57%) of 79 participants. All core community mobilisation tasks were completed in 21 (88%) of 24 of intervention subdistricts. One or more event raising public awareness was held in 23 (96%) of 24 subdistricts. However, a public talk by a participant of CBR and employment facilitation took place in only one subdistrict each, and 17 (71%) of 24 subdistricts had no family support group meetings. The mean ENACTE score was 2·78 (SD 0·19) at baseline and 2·98 (0·02) at 12 months, indicating that on average CBR workers were rated as “done well” across competencies (table 4).

**Table 4:** Intervention fidelity

		**Median (IQR), mean (SD), n (%) or n/N (%)**
**Participant level (n=79)**		
Number of home visits		
	Phase 1	8 (7–9)
	Phase 2	9 (8–11)
	Phase 3	4 (4–5)
	Total	21 (18–25)
Minimum adequate CBR participation (≥10 visits)		71 (90%)
Optimal CBR participation (≥21 visits)		40 (51%)
Months of CBR		12·0 (11·4–12·6)
Continuous receipt of CBR (≥1 home visit/month)		36/77 (47%)
Undertook all core modules		74 (94%)
Number of indicated modules undertaken		5 (4–6)
Started ≥75% indicated modules		65/73 (89%)
Achieved all core (standard) goals		70 (89%)
Number of individual community mobilisation meetings relating to participant		
	0 meetings	34 (43%)
	1 meeting	16 (20%)
	2 meetings	11 (14%)
	≥3 meetings	18 (23%)
**Subdistrict level (n=24)**		
All core community mobilisation tasks completed (resources and leaders identified, awareness raising with leaders and public)		21 (88%)
Number of public awareness raising meetings		
	0 meetings	1 (4%)
	1–2 meetings	6 (25%)
	3–4 meetings	10 (42%)
	5–7 meetings	7 (29%)
Public talk by CBR participant		1 (4%)
Number family support group meetings		
	0 meetings	17 (71%)
	1–2 meetings	3 (13%)
	≥3 meetings	4 (17%)
Employment opportunities identified		2 (8%)
Employment opportunities facilitated		1 (4%)
**CBR worker level (n=11)**		
Externally assessed ENACT score trial baseline		2·78 (0·19)
Externally assessed ENACT score trial at 12 months		2·98 (0·02)

CBR=community-based rehabilitation. ENACT=Enhancing Assessment of Common Therapeutic Factors-Ethiopia. Percentages might not equal 100% due to rounding.

Overall, four (5%) of 79 participants in the CBR plus facility-based care group and nine (10%) of 87 participants in the facility-based care group had at least one serious adverse event (p=0·21; appendix p 4). Serious adverse events included six deaths (no suicides), two suicide attempts, and six hospitalisations. Data collectors were unmasked during the course of 33 (22%) of 153 interviews (26 [35%] of 74 participants in the CBR plus facility-based care group and 7 [9%] of 79 participants in the facility-based care group); all unmasking occurred after 12-month WHODAS data had been collected.

Participants with 12-month data available were more likely to be female (p=0·055), younger age (p=0·040), lower socio-economic status (p=0·032), rural residents (p=0·058), have longer illness duration (p=0·080), fewer relapses (p=0·042), and lower caregiver burden (p=0·024; appendix pp 5–6). There was evidence of effect-modification by alcohol use disorder, with a greater effect on illness severity among participants with alcohol use disorder than without (p value for interaction=0·017; appendix p 6). There was no evidence of effect modification by other variables on WHODAS score or illness severity (appendix pp 6–7). The findings were similar under sensitivity analyses (appendix pp 7–17). At 12 months, the intra-cluster correlation for WHODAS was 0·02 (95% CI 0·00–0·18).

## Discussion

To our knowledge, RISE is the first randomised trial to evaluate the effectiveness of any psychosocial intervention for people with schizophrenia in a low-income country, and it is the first to evaluate a psychosocial intervention as an adjunct to task-shared facility-based care in any setting globally. The inclusion of structured community mobilisation components is unique among randomised evaluations. In this care model, CBR was effective in reducing disability (particularly in relation to participation, social interactions, and cognition), symptom severity, caregiver tension and worrying; and in increasing antipsychotic medication adherence and attendance to facility-based care among people with schizophrenia. However, we found no evidence that CBR impacted on physical restraint, discrimination, employment or work, and household and self-care aspects of disability. The benefits of CBR were only evident after 12 months of the study. Intervention fidelity was good in terms of the number and content of CBR home visits and CBR worker competence.

Findings from the PRIME cohort study showed that access to task-shared facility-based care significantly improved symptom severity, disability, discrimination, and physical restraint over a 12-month period.^11^ Our results demonstrate that a supplementary level of care, CBR, can increase the benefits of facility-based care and produce additional impacts on disability and symptoms in people with schizophrenia who have not responded to or engaged with standard care. We propose that CBR achieved its impact on disability in two ways. First, by maximising engagement with facility-based care; therefore, facilitating the use of antipsychotic medication. Medication adherence could have a positive effect on clinical severity, which might contribute to improved functioning. The strongest intervention effects were seen on facility attendance, medication adherence, and symptoms, suggesting this pathway could have had a prominent role. Descriptive analysis showed that at 12 months a greater proportion of participants in the CBR plus facility-based care group were prescribed medication and had access to free medication than participants in the facility-based care group. We suggest the CBR workers’ success is attributable to a combination of mobilising support for transportation to health care, therefore minimising barriers related to geographical accessibility; obtaining medication fee waivers, which increased affordability; psychoeducation (promoting understanding of the potential advantages of treatment), thereby increasing acceptability; and appointment reminders and timely referral for relapse and medication side-effects, which optimised the adequacy of care.

Second, CBR might shape an individuals’ social environment, indicated by the strongest positive effects on WHODAS domains “getting along” and “participation”. Cognitive performance, which showed weaker positive effects, also predicts community independence.^23^ As the main care providers, family members have a powerful influence on illness experience and social roles. We suggest that, as the pilot study demonstrated,^10^ emphasising human rights facilitated attitudinal shifts within families, which in turn promoted participation. Community mobilisation, including raising awareness and engagement with community members to mobilise support, was generally comprehensive and might have facilitated social inclusion. Pathways to improved functioning are likely to be synergistic; social networks were encouraged to support transportation to, and the costs of, facility-based care. However, the absence of an effect on employment, discrimination, and restraint, suggests that impacts on the wider social environment were partial, which might be explained by the non-implementation of some community activities, such as public talks by participants. The quality of community mobilisation or actual resources mobilised were not assessed. Restrained individuals might reflect a subgroup with refractory illness who require more specialist interventions. The low prevalence of restraint made it challenging to detect an intervention effect; furthermore, improvements in work-related functioning, discrimination, and restraint might require attitude and behaviour changes that emerge beyond 12 months. We anticipate future qualitative analyses will be central to understanding the extent that community resources were actually mobilised, reasons for difficulties, and the impact on recovery.^24^

Although non-significant in sensitivity analyses, the adverse impact of CBR on unemployment deserves scrutiny. Vocational skills training and microfinance are often included in CBR programmes and similar models.^7^ RISE omitted similar livelihood support due to sustainability concerns, instead including optional support to resume farming and identification of employment opportunities. However, employment opportunities are scarce.^10^ Furthermore, employment status is a crude measure of economic impact and household economic status might be a superior measure.

There were missing data on the self-rated WHODAS and BPRSE due to participants being too unwell to respond. Lower power to detect intervention effects might explain the difference with proxy-rated WHODAS and CGI scores, which do not rely on self-reporting. The tendency of people with schizophrenia to overestimate functioning compared with external assessment has also been reported.^16^, ^25^ The lack of effect on the BFS could be because of the large proportion of work-related items.^19^ The absence of impact on caregiver depression is not surprising since caregivers did not receive an evidence-based intervention; however, the effects on worrying and tension demonstrate that the emotional burden of caregiving can be alleviated by an intervention primarily targeting their relative.

Our findings reflect the benefits of community-based psychosocial interventions found in middle-income country settings.^3^, ^6^, ^26^, ^27^, ^28^ The COPSI RCT of collaborative community-based care in India represents the most comparable previous evaluation.^6^ However, reflecting standard care in that context, all COPSI participants received regular psychiatrist reviews and free antipsychotic medication. Our results show that lay-worker delivered psychosocial interventions are also effective when delivered alongside task-shared facility-based care (delivered by non-physicians), as promoted by WHO Mental Health Gap Action Programme, and when free antipsychotic medication is not universally available. Furthermore, despite relying on non-specialist supervisors (a novel feature contrasting with psychiatric social worker supervision in COPSI^6^) no concerns around participant safety or CBR worker competence were identified in this analysis. We have shown that participation rates are high and that CBR is effective in an area highly reliant on time-consuming subsistence farming, where many participants reside in remote areas, and where there are few material resources to access treatment or support recovery. As such, we have demonstrated that CBR is generalisable to low-income country settings, even when modified to match available mental health-care infrastructure.

On the basis of our current findings, we propose a holistic psychosocial intervention, encompassing support with treatment engagement and efforts to shape the social environment. Planned exploratory and qualitative analyses will help to determine the active components of CBR and might allow the intervention to be rationalised. The absence of beneficial effects at 6 months suggests prolonged participation is needed to establish relationships and support meaningful changes in behaviour; therefore, we propose a minimum 12-month intervention is required. We acknowledge that CBR was delivered by a new cadre of workers; as such, the model is not immediately scalable in Ethiopia. However, our study provides an important proof of concept; lay workers after 5 weeks of training and 6 months of work experience can address important aspects of the complex needs of people with schizophrenia. Incorporating mental health into the work of existing community health workers has proved difficult because of their high occupational load;^29^ the use of health extension workers to deliver CBR was discounted early on for this reason.^9^ Increased numbers of generic community health workers might address these difficulties. Alternatively, the development of new cadres of community-based non-specialist workers, including peer supporters, could be key to achieving universal health coverage for people with schizophrenia.^1^, ^24^ Mental health can also be integrated into existing CBR programmes,^30^ although, coverage might be limited.

Study limitations include the lack of assessment for an enduring impact of CBR after the intervention had terminated and the relatively large number of outcomes. Each of these was prespecified in the analysis plan because of the potential association with the intervention; and we have been conservative in our interpretation. However, it is possible that some of these associations arose due to chance. Outcomes measured in community members—eg, discrimination—would help to elucidate the utility of community mobilisation. Finally, the resource implications of CBR are currently unknown.

In conclusion, we have shown that psychosocial support can promote functional recovery of people with schizophrenia in a low-income country and demonstrated the feasibility of lay worker delivery. Further research should investigate large-scale implementation of models such as this.

## Data sharing

Anonymised participant data and a data dictionary will be made available by 12 months after publication. Data will be shared following a reasonable submitted request and approval by the corresponding author. The study protocol and statistical analysis are publicly available.^12^

## Declaration of interests

CH reports support from the National Institute of Health Research (NIHR) through a RIGHT grant (NIHR200842) and the NIHR Global Health Research Unit on Health System Strengthening in sub-Saharan Africa, King's College London (GHRU 16/136/54), using aid from the UK Government; and support from African Mental Health Research Initiative as part of the Developing Excellence in Leadership, Training, and Science Africa Initiative (DEL-15–01). HAW reports support from the UK Medical Research Council (MRC) and the UK Department for International Development (DFID) under the MRC-DFID concordat agreement, which is also part of the European and Developing Countries Clinical Trials Partnership programme 2 supported by the EU (MR/R010161/1). All other authors declare no competing interests. The views expressed in this publication are those of the authors and not necessarily those of the NIHR or the Department of Health and Social Care.
