# Correction to *Lancet Glob Health* 2022; 10: e530–42

**DOI:** 10.1016/S2214-109X(22)00216-9

**Published:** 2022-05-10

*Asher L, Birhane R, Weiss HA, et al. Community-based rehabilitation intervention for people with schizophrenia in Ethiopia (RISE): results of a 12-month cluster-randomised controlled trial.* Lancet Glob Health *2022; **10:** e530–42*—In this Article the acknowledgments have been corrected to add “MDS was not employed by the Wellcome Trust at the time of the study.” This correction has been made as of May 10, 2022.

