# Supplementary material for: Community-based rehabilitation intervention for people with schizophrenia in Ethiopia (RISE): results of a 12-month cluster-randomised controlled trial
Source: Lancet Glob Health. 2022 Mar 15;10(4):e530–42. doi: 10.1016/S2214-109X(22)00027-4 (PMC8938762; doi:10.1016/S2214-109X(22)00027-4)
Supplement: Supplementary appendix [file mmc1.pdf]

THE LANCET
Global Health

Supplementary appendix

This appendix formed part of the original submission and has been peer reviewed. We post it as supplied by the authors.

Supplement to: Asher L, Birhane R, Weiss HA, et al. Community-based rehabilitation intervention for people with schizophrenia in Ethiopia (RISE): results of a 12-month cluster-randomised controlled trial. *Lancet Glob Health* 2022; **10:** e530–42.

Supplementary material

Table 1 Minor amendments to published protocol

Item	Details	Reason for change
**Data collection window**	Data collection windows specified as 12 months +/10 weeks. Primary analysis will include participants with data collected in window and sensitivity analyses for broader time window e.g. +/6 weeks and data collected at any time.	Data collection window was not specified in published protocol.
**Relapse measurement**	1. Relapse operationalized as either physical restraint or deterioration in illness course, measured using LCS, since previous data collection point<br>2. LIFE chart not used as outcome measure	Concerns re completeness and validity of LIFE chart
**Health service use measurement**	Additional items on access to free medication, included as exploratory outcome measure 12 months	Access to medication identified as important component of health service use in pilot phase
**Economic activity measure**	Income not used as measure of economic activity. % Unemployed/ not earning and % hunger due to lack of resources used.	Concerns re completeness and validity of income data
**Social support measure**	OSLO social support measure used as potential moderator not secondary outcome.	Secondary outcomes rationalised to include only key outcomes of interest
**Health service use for physical complaints**	6 and 12 month health service use for physical complaints are not included as exploratory/secondary outcomes due to very low numbers reporting this outcome	Secondary outcomes rationalised to include only key outcomes of interest
**Caregiver-rated person with schizophrenia medication adherence**	Caregiver reported person with schizophrenia medication adherence will be used only for items missing from person with schizophrenia-reported scales.	Secondary outcomes rationalised to include only key outcomes of interest.
**Alcohol use disorder**	6 and 12 month person with schizophrenia alcohol use disorder (AUDIT) included as exploratory outcomes (for subsequent analysis) not secondary outcomes	Secondary outcomes rationalised to include only key outcomes of interest
**Nutritional status**	6 and 12 month nutritional status included as exploratory outcomes (for subsequent analysis) not secondary outcomes	Secondary outcomes rationalised to include only key outcomes of interest
**Caregiver stigma**	6 and 12 month caregiver stigma included as exploratory outcomes (for subsequent analysis) not secondary outcomes	Secondary outcomes rationalised to include only key outcomes of interest
**Caregiver economic activity**	6 and 12 month caregiver economic activity included as exploratory outcomes (for subsequent analysis) not secondary outcomes	Secondary outcomes rationalised to include only key outcomes of interest
**Depression (person with schizophrenia)**	6 and 12 month depression (PHQ-9) in the person with schizophrenia added as exploratory outcomes (for subsequent analysis).	PH9-9/ depression used as a measure of 'self esteem and hope', which was identified as a potentially important outcome in the piloting phase.
**6 month exploratory outcomes**	All 6 month outcomes, with the exception of disability (proxy reported WHODAS) and symptoms (Clinical Global Impression), will be considered as exploratory outcomes (for subsequent analysis) rather than secondary outcomes. Six month exploratory outcomes therefore include self-reported WHODAS, Butarjira functioning scales, BPRSE, relapse, health service use (mental), physical restraint, discrimination, economic activity (person with schizophrenia and caregiver), medication adherence, caregiver depression and caregiver burden	Secondary outcomes rationalised to include only key outcomes of interest
**CBR worker competency assessment by supervisor and self assessment (process data)**	Omitted from process data analysis. ENACT assessment retained.	Concerns re validity of supervisor reported and self reported measures.
**Baseline characteristics of participants who did and did not consent**	Not compared	Only 1 of 167 eligible participants did not consent
**Baseline characteristics of subdistricts**	Added comparison between treatment arms and lost to follow up	To determine comparability of treatment arms
**Primary analysis**	Health centre will be adjusted for as a fixed effect.<br>CBR worker will not be adjusted for as a random effect.	Randomisation was stratified by health centre.<br>There was high collinearity between CBR worker and health centre.

**Primary analysis**	The primary analysis will be complete case, adjusting for variables imbalanced at baseline and associated with missing outcome data. We will complete a sensitivity analysis using multiple imputation.	Minimal missing data for primary outcome
**Primary analysis**	6 and 12 month time points will be analysed separately.	We hypothesize a different intervention effect due to the different intensity of the intervention over the 12 month period.
**Sensitivity analysis**	Complier average causal effect (CACE) analysis removed as a sensitivity analysis	Comparable trials have found that participants attending less sessions have better outcomes. CACE analysis is therefore not appropriate as this assumes the opposite.

Table 2 Outcome measures

Outcome	Measure	Timing
**Person with schizophrenia outcomes (lay data collector administered interview)**		
**Disability**	**Proxy-rated 36-item WHODAS 2.0 total score** (1)	6 & 12 months (primary outcome)
	**Proxy-rated 36-item WHODAS 2.0 domain scores**	6 & 12 months
	**Proxy-rated WHODAS 2.0 number of days unable to work in last month**	6 & 12 months
	**Self-rated 36-item WHODAS 2.0 total score**	12 months
	**Self-rated 36-item WHODAS 2.0 domain scores**	12 months
	**Self-rated WHODAS 2.0 number of days unable to work in last month**	12 months
	**Butajira Functioning Scale (BFS) total score (2)**<br>A validated indigenous functioning scale, specific to persons with severe mental disorders in the Ethiopian context. 33 items applicable to both men and women rated 1 (no difficulty) to 5 (cannot do task). Total range 33 to 165.	12 months
**Relapse**	Relapse is defined as either of:<br>- Report of physical restraint at 12 month assessment by either the person with schizophrenia or caregiver<br>- **Life chart schedule (LSC)** (3) course type question indicates a deterioration between 6 months and 12 months, defined as follows:<br>o Score of 4 (never psychotic in preceding 6 months) changes to 1 (episodic) or 2 (continuous illness)<br>o Score of 1 (episodic) changes to 2 (continuous)	12 months
**Health service use**	**Adapted Client Service Receipt Inventory (CSRI)** (4, 5**).**<br>Used to derive binary question: % with no health facility attendances (at health centre or outpatient facility) for any mental health problem (including psychosis, depression or substance use) in the last 3 months	12 months
**Antipsychotic medication adherence (frequency)**	5 point ordinal scale assessing medication adherence over the last one month. Binarised to all the time/ most of the time vs sometimes/occasionally/never. Scale developed for COPSI trial (6, 7)	12 months
**Antipsychotic medication adherence (behaviours)**	4-item Morisky Medication Adherence Scale. Binarised to no non-adherent behaviour vs any non-adherent behaviour. Scale used in COPSI trial (6, 7).	12 months
**Physical restraint**	Self or caregiver reported physical restraint in the last 6 months- binary question developed for PRIME.	12 months
**Discrimination**	**Section 1 (unfair treatment) of Discrimination and Stigma Scale-12 (DISC-12)** (8)<br>21 items asking the participant if they have experienced different types of discrimination relating to their mental illness. All items are rated on a 4-point likert scale. Binarised to any experience of discrimination (1 (a little), 2 (moderately) or 3 (a lot) on any item)	12 months
**Economic activity**	Question on employment status developed for PRIME. Binarised to employed (fulltime/part time or paid/self employment) and unemployed (voluntary employment, unemployed (including housewife), student and retired).	12 months
**Person with schizophrenia outcomes (clinician administered interview)**		
**Symptom severity**	**Clinical Global Impression (CGI)- severity** (9).<br>Rated on a seven point score ranging from 1, when the person with schizophrenia is assessed to have no illness, to 7, when the person with schizophrenia is among the most severely ill. The scale is completed entirely based on clinical judgment.	6 & 12 months
	**Brief Psychiatric Rating Scale- Expanded version (BPRS-E)** (10).<br>Focuses on symptoms of psychosis, but also has items covering the symptom domains of somatic concerns, anxiety, depression and mania. Each symptom is rated along a seven point severity continuum (1 to 7). Total score is obtained by adding the ratings for each of the 24 items. Range is 24 to 168 with higher score indicating greater severity. Individual BPRS-E items and total score are sensitive to change in persons with persistent schizophrenia (11). The scale has been previously used in Ethiopia (12) and has good inter-rater reliability (>0.8 comparing psychiatric nurses and	12 months

	psychiatrists) in this setting (personal communication, Dr Charlotte Hanlon)(13). Inter-rater and test-retest reliability as well as internal consistency are also high in high-income settings (14).	
**Caregiver outcomes (lay data collector administered interview)**		
**Caregiver depression**	**Patient Health Questionnaire- 9 (PHQ-9) [78].** Incorporates DSM-IV depression diagnostic criteria with other leading major depressive symptoms into a 9-item self-report tool. Each item rated as '0' (not at all) to '3' (nearly every day); possible range 0 to 27 with higher scores indicating greater severity. Shown to be valid in the Ethiopian setting. Reported mean PHQ score and % PHQ ≥5.	12 months
**Caregiver burden**	**Involvement evaluation questionnaire (IEQ)** -Caregiving consequences section (15). 31-item questionnaire assessing aspects of caregiver burden. All items are scored on 5-point Likert scales (0 never to 4 always). Domain scores can be computed (tension & urging range 0 to 36; worrying & supervision range 0 to 24).	12 months
	Binary question developed for PRIME on whether family/friends have stopped work because of the person with schizophrenia's ill health	12 months

Table 3 Anti-psychotic medication prescription and free medication access by arm

	Facility-based care group	CBR plus facility-based care group	Total
**Antipsychotic medication prescription**			
**Baseline**	n=87	n=79	n=166
**No anti-psychotic medication**	49 (56.3%)	37 (46.8%)	86 (51.8%)
**Any anti-psychotic medication**	38 (43.7%)	42 (53.7%)	80 (48.2%)
**6 months**	n=81	n=70	n=151
**No anti-psychotic medication**	32 (39.5%)	22 (31.4%)	54 (35.8%)
**Any anti-psychotic medication**	49 (60.5%)	48 (68.6%)	97 (64.2%)
**12 months**	n=79	n=75	n=154
**No anti-psychotic medication**	40 (50.6%)	17 (22.7%)	57 (37.0%)
**Any anti-psychotic medication**	39 (49.4%)	58 (77.3%)	97 (63.0%)
**Access to free anti-psychotic medication**			
**12 months**	n=79	n=74	n=153
**No free medication**	55 (69.6%)	37 (50.0%)	92 (60.1%)
**Free medication**	24 (30.4%)	37 (50.0%)	61 (39.9%)

Table 4 Name of prescribed anti-psychotic medication

	Facility-based care group	CBR plus facility-based care group	Total
**Baseline (n=166)**			
**None**	49 (56.3%)	37 (46.8%)	86 (51.8%)
**Oral haloperidol**	13 (14.9%)	12 (15.2%)	25 (15.1%)
**Oral chlorpromazine**	22 (25.3%)	29 (36.7%)	51 (30.7%)
**Oral risperidone**	0 (0)	1 (1.3%)	1 (0.6%)
**Depot fluphenazine +/- any oral medication**	3 (3.5%)	0 (0)	3 (1.8%)
**6 months (n=151)**			
**None**	32 (39.5%)	22 (31.4%)	54 (35.7%)
**Oral haloperidol**	20 (24.7%)	14 (20.0%)	34 (22.5%)
**Oral chlorpromazine**	27 (33.3%)	31 (44.3%)	58 (38.4%)
**Oral risperidone**	0 (0)	1 (1.4%)	1 (0.7%)
**Depot fluphenazine +/- any oral medication**	2 (2.5%)	2 (2.9%)	4 (2.7%)
**12 months (n=154)**			
**None**	40 (50.6%)	17 (22.7%)	57 (37.0%)
**Oral haloperidol**	18 (22.8%)	15 (20.0%)	33 (21.4%)
**Oral chlorpromazine**	18 (22.8%)	30 (40.0%)	48 (31.2%)
**Oral risperidone**	0 (0)	3 (4.0%)	3 (2.0%)
**Depot fluphenazine +/- any oral medication**	3 (3.8%)	10 (13.3%)	13 (8.4%)

Table 5 Secondary outcomes in persons with schizophrenia at 6 months (+/- 10 weeks)

Outcome	Facility-based care group (n=60)	CBR plus facility-based care group (n=52)	Minimally adjusted mean difference or odds ratio (95% CI)[a]	P value	Fully adjusted mean difference or odds ratio (95% CI)[b]	p value	Effect size (95% CI)
**Functioning**							
**Proxy-reported WHODAS total score (mean [SD])**	45.4 (26.6)	44.0 (24.2)	-0.50 (-9.78,8.79)	0.92	-0.48 (-11.98,11.02)	0.94	0.02 (-0.35, 0.39)
**Proxy-reported WHODAS domain scores (mean [SD])**							
Cognition	51.7 (35.3)	51.1 (31.1)	1.66 (-10.59,13.91)	0.79	4.22 (-11.09,19.53)	0.59	0.13 (-0.24, 0.50)
Mobility	22.8 (25.9)	21.3 (25.3)	-1.89 (-12.02,8.24)	0.71	-1.05 (-12.74,10.64)	0.86	0.04 (-0.33, 0.41)
Self care	34.8 (29.2)	33.8 (28.6)	-1.88 (-13.52,9.76)	0.75	-2.01 (-15.70,11.68)	0.77	0.07 (-0.30, 0.44)
Getting along	44.0 (34.6)	47.4 (34.0)	5.91 (-6.91,18.7)	0.37	4.12 (-10.33,18.58)	0.58	0.12 (-0.25, 0.49)
Life activities: household	65.7 (35.7)	62.9 (32.2)	-1.53 (-13.90,10.83)	0.81	-1.00 (-14.64,12.64)	0.89	0.03 (-0.34, 0.40)
Life activities: work	61.1 (35.3)	59.8 (31.7)	0.32 (-11.93, 12.58)	0.96	0.30 (-13.05, 13.65)	0.97	0.009 (-0.36, 0.38)
Participation	42.4 (26.8)	40.5 (23.2)	-2.27 (-12.08,7.54)	0.65	-2.07 (-12.77,8.64)	0.73	0.08 (-0.29, 0.45)
**Proxy-reported number of days unable work last month (Median [IQR])**	8 (2,30)	6.5 (2,20)	-0.89 (-5.56,3.77)	0.71	-1.05 (-6.61,4.51)	0.71	0.09 (-0.28, 0.46)
**Symptom severity**							
**At least mildly ill (CGI score ≥3) (n [%])** (n=103)	52 (86.7%)	42 (80.8%)	OR 0.75 (0.23, 2.45)	0.64	OR 0.69 (0.18, 2.73)[c]	0.60	-

[a] Adjusted for sub-district (cluster) as random effect and health centre and baseline score of outcome as fixed effects
[b] Adjusted for sub-district (cluster) as a random effect and health centre, baseline score of outcome, baseline disability (proxy-reported total WHODAS), sex, baseline socio-economic status, baseline employment status, travel time to health facility, baseline caregiver employment status, baseline social support, and baseline caregiver depression (PHQ-9) as fixed effects. Social support reduced to two categories to avoid problems with data sparsity.
[c] Fully adjusted model excludes travel time to health facility due to data sparsity

Table 6 Serious adverse events by treatment arm and overall

Serious adverse event type	Facility-based care group (n=87)	CBR plus facility-based care group (n=79)	P value [a]	All (n=166)
**Death due to suicide**	0 (0)	0 (0)	-	0 (0)
**Death due to cause except suicide**	4 (4.6%)	2 (2.5%)	0.48	6 (3.6%)
**Suicide attempt**	1 (1.2%)	1 (1.3%)	0.95	2 (1.2%)
**Hospitalisation due to suicide attempt or serious side effect of anti-psychotic medication**	0 (0)	0 (0)	-	0 (0)
**Hospitalisation due any other serious medical emergency**	4 (4.6%)	2 (2.5%)	0.48	6 (3.6%)
**Participants with any SAE (all types)**	9 (10.3%)	4 (5.1%)[b]	0.21	13 (7.8%)

[a] Chi^2 test [b] One participant experienced 2 SAEs

Table 7 Baseline characteristics of completers of outcome evaluation and those lost to follow up at 12 months (+/-10 weeks)

	**Lost before 12 month evaluation (n=17)**	**Completed 12 month outcome evaluation (n=149)**	**P value[a]**
**Persons with schizophrenia**			
**Sex (n [%])**			
Male	14 (13.6%)	89 (86.4%)	0.055
Female	3 (4.8%)	60 (95.2%)	
**Age (years) (n [%])**			
Less than 25	4 (8.2%)	45 (91.8%)	0.040
26 to 35	2 (3.8%)	50 (96.2%)	
36 or more	11 (16.9%)	54 (83.1%)	
**Marital status** [b] (n [%]) (n=155)			
Single	6 (7.6%)	73 (92.4%)	0.40
Has a partner (married, married not living together)	7 (13.7%)	44 (86.3%)	
Separated/divorced/widowed	4 (16%)	21 (84.0%)	
**Occupation** (n [%])			
No occupation	3 (12.5%)	21 (87.5%)	0.62
Home worker	5 (8.3%)	55 (91.7%)	
Unskilled labourer	8 (10.5%)	68 (89.5%)	
Other	1 (16.7%)	5 (83.3%)	
**Education status** [b] **(n [%])** (n=155)			
No formal education	7 (8.5%)	75 (91.5%)	0.28
Primary education	7 (12.3%)	50 (87.7%)	
Secondary education and above	3 (18.8%)	13 (81.3%)	
**Socio economic status** [b] **(n [%])** (n=154)			
Higher (poverty index ≤ 3)	14 (15.7%)	75 (84.3%)	0.032
Lower (poverty index >3)	3 (4.6%)	62 (95.4%)	
**Residence** [b] **(n [%]) (n=154)**			
Urban	3 (15.8%)	16 (84.2%)	0.058
Rural	14 (10.4%)	121 (89.6%)	
**Travel time to nearest health facility** [b] **(n [%])** (n=155)			
≤ 60 minutes	11 (11.1%)	88 (88.9%)	0.65
61 to 120 minutes	2 (6.7%)	28 (93.3%)	
≥121 minutes	4 (15.4%)	22 (84.6%)	
**Diagnosis**			
Schizophrenia	14 (10.1%)	124 (89.9%)	0.87
Schizoaffective/ schizophreniform disorder	3 (10.7%)	25 (89.3%)	
**Duration illness** [b] **(median [IQR])** (n=119)	1.8 (1.0, 8.3)	4.0 (2.0, 9.0)	0.080
**Co-morbid medical disorder** [b] **(n [%])** (n=148)			
No	11 (8.0%)	127 (92.0)	0.17
Yes	3 (30.0%)	7 (70.0%)	
**Proxy-reported total WHODAS (mean [SD])**	58.1 (23.9)	50.7 (23.5)	0.15
**Symptom severity BPRS-E total (mean [SD])** (n=160)	51.9 (12.1)	47.5 (13.9)	0.14
**Illness severity CGI (n [%])**			
Normal or borderline (score <3)	0 (0)	8 (100%)	0.41[c]
At least mildly ill (score ≥3)	17 (10.8%)	141 (89.2%)	
**Illness course last 6 months (LCS) (n [%])**			
Episodic	2 (28.6%)	5 (71.4%)	0.042[c]
Continuous	15 (11.4%)	117 (88.6%)	
Never psychotic	0 (0)	27 (100%)	
**Antipsychotic medication adherence (n [%])** (n=160)			
All or most of time	7 (9.9%)	64 (90.1%)	0.55
Sometimes, occasionally or not at all	10 (11.2%)	79 (88.8%)	
**Engagement with care (n [%])** (n=160)			
No healthcare attendance and no medication adherence	5 (8.1%)	57 (91.9%)	0.27
Either healthcare attendance or medication adherence	6 (15.0%)	34 (85.0%)	
Healthcare attendance and medication adherence	6 (10.3%)	52 (89.7%)	
**AUDIT total (n [%])** (n=160)			
< 8 (no alcohol use disorder)	11 (8.6%)	117 (91.4%)	0.12
≥8 (alcohol use disorder)	6 (18.6%)	26 (81.3%)	

**Restrained last 6 months (n [%])**			
No	15 (9.6%)	142 (90.5%)	0.34
Yes	2 (22.2%)	7 (77.8%)	
**Any experience of discrimination last 6 months (n [%])**			
No	6 (7.7%)	72 (92.3%)	0.16
Yes	11 (12.5%)	77 (87.5%)	
**Unemployed (n [%])**			
No	9 (12.9%)	61 (87.1%)	0.36
Yes	8 (8.3%)	88 (91.7%)	
**Social support (n [%])**			
Poor	3 (5.2%)	55 (94.8%)	0.30
Intermediate	10 (12.5%)	70 (87.5%)	
Strong	4 (14.3%)	24 (85.7%)	
**Caregiver**			
**Caregiver burden Total IEQ (mean [SD])**	49.5 (16.0)	39.3 (17.6)	0.024
**Caregiver depression (n [%])**			
No (PHQ < 5)	11 (11.8%)	82 (88.2%)	0.56
Yes (PHQ-9 ≥ 5)	6 (8.2%)	67 (91.8%)	
**Caregiver unemployment (n [%])**			
No	13 (13.3%)	85 (86.7%)	0.20
Yes	4 (5.9%)	64 (94.1%)	
**Sub-districts**	**Lost before 12 month evaluation (n=2)**	**Completed 12 month outcome evaluation (n=46)**	
**Location (n [%])**			
Urban	0 (0)	3 (100%)	0.88[c]
Rural	2 (4.4%)	43 (95.6%)	
**Baseline number of participants** (median [IQR])	1 (1, 1)	3 (2, 5)	0.070[d]
**Proxy-reported total WHODAS** (median [IQR])	43.9 (17.9, 69.8)	54.1 (44.6, 61.6)	0.66[a]

[a]By logistic regression adjusting for subdistrict (cluster) as a random effect and adjusting for health centre as a fixed effect, unless otherwise stated.
[b]Data collected at PRIME baseline
[c]By Chi^2 test due to data sparsity
[d] By Mann-Whitney test due to data sparsity

Table 8 Subgroup (moderator) analysis for 12 month (+/- 10 weeks) illness severity (CGI)

**Outcome**	**N**	**At least mildly ill (CGI ≥3) n (%)**		**Fully adjusted OR[a] (95% CI)**	**P value**	**p value for interaction**
		**Facility-based care group**	**CBR plus facility-based care group**			
**Gender**						
**Men**	89	35 (81.4%)	31 (67.4%)	0.26 (0.06, 1.03)	0.055	0.94
**Women**	60	28 (84.9%)	17 (63.0%)	0.27 (0.05, 1.51)	0.14	
**Baseline symptom severity**						
**Total BPRS-E score < median**	75	31 (75.6%)	21 (61.8%)	0.46 (0.11, 1.94)	0.29	0.28
**Total BPRS-E score ≥ median**	74	32 (91.4%)	27 (69.2%)	0.13 (0.02, 0.78)	0.025	
**Baseline functioning**						
**Total proxy-reported WHODAS score < median**	77	29 (72.5%)	19 (51.4%)	0.28 (0.07, 1.14)	0.077	0.75
**Total proxy-reported WHODAS score ≥ median**	72	34 (94.4%)	29 (80.6%)	0.19 (0.03, 1.34)	0.096	
**Baseline alcohol use disorder (n=143)**						
**AUDIT score <8**	117	50 (80.7%)	36 (65.5%)	0.59 (0.15, 2.32)	0.45	0.017
**AUDIT score ≥8**	26	12 (92.3%)	10 (76.9%)	0.01 (0.0003, 0.30)	0.0080	
**Baseline social support**						

**Strong/ intermediate support (OSLO)**	95	47 (85.5%)	26 (65%)	0.42 (0.10, 1.78)	0.24	0.34
**Poor support (OSLO)**	54	16 (76.2%)	22 (66.7%)	0.14 (0.03, 0.82)	0.030	
**Baseline socio-economic status (n=137)**						
**Higher (poverty index ≤ 3)**	75	29 (85.3%)	28 (68.3%)	0.39 (0.08, 1.81)	0.23	0.31
**Lower (poverty index >3)**	62	29 (82.9%)	17 (63.0%)	0.12 (0.02, 0.73)	0.022	

[a] Adjusted for sub-district (cluster) as a random effect and health centre, baseline score of outcome, baseline disability (proxy-reported total WHODAS), sex, age, baseline socio-economic status, baseline illness course, baseline caregiver burden (IEQ), illness duration, baseline employment status, baseline caregiver employment status, baseline social support, and baseline caregiver depression (PHQ-9) as fixed effects. Illness course and social support reduced to two categories to avoid problems with data sparsity.

Table 9 Subgroup (moderator) analysis for 12 month (+/- 10 weeks) proxy-reported WHODAS total score

Outcome	N	Facility-based care group (n=76)	CBR plus facility-based care group (n=73)	Fully adjusted mean difference[a] (95% CI)	P value	p value for interaction
**Gender**						
**Men**	89	44.8 (22.5)	43.9 (20.8)	-6.25 (-15.19,2.70)	0.17	0.42
**Women**	60	47.8 (24.4)	34.9 (24.6)	-11.86 (-23.04, -0.67)	0.038	
**Baseline symptom severity**						
**Total BPRS-E score < median**	75	42.3 (22.3)	33.9 (24.4)	-10.46 (-20.71, -0.21)	0.046	0.58
**Total BPRS-E score ≥ median**	74	50.5 (23.9)	46.4 (19.2)	-6.61 (-16.32,3.11)	0.18	
**Baseline functioning**						
**Total proxy-reported WHODAS score < median**	76	36.6 (22.4)	29.5 (20.4)	-7.28 (-18.26,3.71)	0.19	0.86
**Total proxy-reported WHODAS score ≥ median**	73	56.7 (19.5)	50.8 (19.5)	-8.65 (-19.80,2.50)	0.13	
**Baseline alcohol use disorder (n=143)**						
**AUDIT score <8**	117	47.5 (24.0)	40.5 (22.2)	-10.03 (-18.56,-1.48)	0.021	0.32
**AUDIT score ≥8**	26	40.1 (20.3)	42.7 (23.3)	-0.89 (-17.28, 15.49)	0.92	
**Baseline social support**						
**Strong/ intermediate support (OSLO)**	94	46.1 (25.0)	38.5 (22.2)	-7.48 (-17.30,2.34)	0.14	0.82
**Poor support (OSLO)**	55	46.1 (18.6)	43.0 (22.9)	-9.25 (-20.91,2.34)	0.12	
**Baseline socio-economic status (n=137)**						
**Higher (poverty index ≤ 3)**	75	43.1 (23.6)	40.9 (20.8)	-7.19 (-16.67,2.28)	0.14	0.86
**Lower (poverty index >3)**	62	49.5 (22.0)	43.5 (24.7)	-8.37 (-18.88,2.13)	0.12	

[a] Adjusted for sub-district (cluster) as a random effect and health centre, baseline score of outcome, baseline disability (proxy-reported total WHODAS), sex, age, residence, baseline socio-economic status, baseline illness course, baseline caregiver burden (IEQ), illness duration, baseline employment status, baseline caregiver employment status, baseline social support, and baseline caregiver depression (PHQ-9) as fixed effects. Illness course and social support reduced to two categories to avoid problems with data sparsity.

Table 10 Primary and secondary outcomes in persons with schizophrenia at 12 months (+/- 6 weeks)

Outcome	Facility-based care group (n=68)	CBR plus facility-based care group (n=68)	Minimally adjusted mean difference or odds ratio (95% CI)[a]	P value	Fully adjusted mean difference or odds ratio (95% CI)[b]	p value	Effect size (95% CI)
**Primary outcome**							
**Proxy-reported WHODAS-36 total score (mean [SD]) (n=130)**	46.1 (23.7)	42.5 (22.2)	-6.70 (-14.67, 1.28)	0.10	-9.12 (-17.56, -0.68)	0.034	0.39 (0.04, 0.74)
**Secondary outcomes**							
**Functioning**							
**Proxy-reported WHODAS-36 domain scores (mean [SD]) (n=130)**							
Cognition	54.0 (30.6)	49.0 (29.9)	-7.45 (-16.99, 2.10)	0.13	-10.54 (-21.22, 0.15)	0.053	0.34 (0.003, 0.69)
Mobility	21.7 (24.3)	19.6 (21.7)	-3.08 (-12.76, 6.60)	0.53	-2.62 (-14.70, 9.45)	0.67	0.11 (-0.22, 0.46)
Self care	33.0 (28.6)	32.1 (24.8)	-4.57 (-15.37, 6.22)	0.41	-7.20 (-16.66, 2.26)	0.14	0.27 (-0.08, 0.61)
Getting along	49.9 (31.2)	41.4 (31.0)	-13.87 (-24.17, -3.57)	0.0080	-17.31 (-28.09, -6.52)	0.0020	0.53 (0.18, 0.88)
Life activities: household	66.6 (34.8)	64.8 (33.7)	-4.29 (-16.8, 8.22)	0.50	-6.41 (-19.72, 6.90)	0.35	0.19 (-0.16, 0.53)
Life activities: work	62.5 (33.4)	59.7 (31.5)	-4.78 (-16.29, 6.73)	0.42	-7.84 (-21.18, 5.50)	0.25	0.24 (-0.10, 0.59)
Participation	41.2 (23.7)	37.7 (24.8)	-5.48 (-14.07, 3.10)	0.21	-9.68 (-18.84, -0.52)	0.038	0.39 (0.04, 0.74)
**Proxy-rated number of days unable work last month (Median [IQR]) (n=130)**	7 (2, 20)	7 (3, 15)	-2.32 (-6.02, 1.38)	0.22	-4.04 (-8.05, -0.03)	0.048	0.39 (0.04, 0.73)
**Self-rated WHODAS total score (mean [SD]) (n=105)**	34.9 (24.7)	30.5 (19.9)	-5.25 (-15.41, 4.92)	0.31	-6.04 (-15.98, 3.90)	0.23	0.27 (-0.12, 0.65)
**Self-rated WHODAS domain scores (mean [SD]) (n=105)**							
Cognition	36.2 (30.3)	33.9 (27.1)	-1.56 (-15.12, 12.00)	0.82	-3.42 (-16.7, 9.84)	0.61	0.12 (-0.26, 0.50)
Mobility	14.9 (21.4)	17.5 (21.8)	-0.28 (-10.83, 10.26)	0.96	1.87 (-7.59, 11.34)	0.70	0.09 (-0.47, 0.30)
Self-care	22.2 (24.9)	20.4 (18.7)	-2.36 (-13.96, 9.24)	0.69	-3.75 (-13.74, 6.24)	0.46	0.17 (-0.21, 0.56)
Getting along	36.2 (31.5)	25.2 (24.7)	-9.40 (-20.78, 1.97)	0.11	-9.43 (-21.90, 3.03)	0.14	0.33 (-0.06, 0.71)
Life activities: household	56.2 (38.3)	48.2 (34.3)	-8.93 (-23.35, 5.50)	0.23	-13.11 (-28.34, 2.12)	0.092	0.36 (-0.03, 0.74)
Life activities: work	50.3 (35.0)	44.3 (32.4)	-7.14 (-19.82, 5.53)	0.27	-8.59 (-22.63, 5.45)	0.23	0.25 (-0.13, 0.64)

Participation	33.8 (24.1)	28.0 (21.2)	-7.26 (-16.15, 1.63)	0.11	-7.17 (-18.86, 4.51)	0.23	0.31 (-0.07, 0.70)
**Self-rated number of days unable work last month (Median [IQR]) (n=105)**	3.5 (0,10)	5 (0, 10)	-2.39 (-5.56, 0.77)	0.14	-2.69 (-6.71, 1.34)	0.19	0.34 (-0.05, 0.72)
**Proxy-rated BFS (mean [SD]) (n=130)**	98.86 (39.25)	91.14 (35.68)	-8.93 (-22.65, 4.78)	0.20	-9.42 (-22.69, 3.85)	0.16	0.25 (-0.10, -0.59)
**Symptom severity**							
**BPRS-E score (mean [SD]) (SD)** (n=126)	45.32 (14.07)	41.70 (15.70)	-4.78 (-10.35, 0.79) [c]	0.093	-4.98 (-11.13, 1.17) [c]	0.11	0.33 (-0.03, 0.68)
**At least mildly ill (CGI score ≥3) (n [%])** (n=139)	57 (81.4%)	46 (66.7%)	0.42 (0.18, 0.99)	0.046	0.24 (0.08, 0.78)[d]	0.017	-
**Relapse**							
**Relapsed (n [%])** (n=128)	10 (15.4%)	17 (27.0%)	1.56 (0.56, 4.40)[e]	0.40	2.47 (0.74, 8.16)[e]	0.14	-
**Health service use**							
**Non-adherent (takes medication sometimes/ occasionally/ never) (n [%])**	37 (54.4%)	18 (26.5%)	0.19 (0.06, 0.56)	0.0030	0.18 (0.06, 0.51)[d]	0.0010	-
**Any non- adherent behaviour (n [%])**	41 (60.3%)	31 (45.6%)	0.51 (0.23, 1.13)	0.10	0.62 (0.24, 1.57)[d]	0.31	-
**No attendance at health facility for mental health last 3 months (n [%])**	33 (48.5%)	12 (17.6%)	0.21 (0.09, 0.51)	0.0010	0.17 (0.06, 0.49)[d]	0.0010	-
**Physical restraint**							
**Restrained last 6 months (n [%])** (n=136)	4 (5.9%)	5 (7.4%)	1.16 (0.22, 6.21)[f]	0.86	1.98 (0.24, 16.57) [f]	0.53	-
**Discrimination**							
**Any experience of discrimination last 6 months (n [%])**	34 (50%)	43 (63.2%)	2.02 (0.92, 4.45)	0.080	1.77 (0.70, 4.48)[d]	0.23	-
**Economic activity**							
**Unemployed (n [%])**	43 (63.2%)	47 (69.1%)	1.29 (0.48, 3.43)	0.61	2.50 (0.70, 8.87)[d]	0.16	-

[a] Adjusted for sub-district (cluster) as random effect and health centre and baseline score of outcome as fixed effects
[b] Adjusted for sub-district (cluster) as a random effect and health centre, baseline score of outcome, baseline disability (proxy-reported total WHODAS), sex, baseline socio-economic status, baseline illness course, illness duration, baseline employment status, baseline caregiver employment status, baseline social support, baseline marital status, baseline treatment engagement, baseline discrimination and baseline caregiver depression (PHQ-9) as fixed effects. Illness course, illness duration and social support reduced to two categories to avoid problems with data sparsity.
[c] n= 123 due to baseline BPRSE missing data (n=3)
[d] Fully adjusted model excludes baseline marital status due to data sparsity
[e] Fully adjusted model excludes baseline marital status, treatment engagement and socio-economic status due to data sparsity. n=111 in minimally adjusted and fully adjusted models due to data sparsity in health centre categories.
[f] Fully adjusted model excludes baseline marital status, treatment engagement and socio-economic status due to data sparsity. n=95 in minimally adjusted and fully adjusted models due to data sparsity in health centre categories.

Table 11 Secondary outcomes in caregivers at 12 months (+/- 6 weeks)

Outcome	Facility-based care group (n=64)	CBR plus facility-based care group (n=66)	Minimally adjusted mean difference or odds ratio (95% CI)[a]	P value	Fully adjusted mean difference or odds ratio (95% CI)[b]	p value	Effect size (95% CI)
**Caregiver depression**							
**PHQ-9 score (mean [SD])**	4.53 (3.19)	4.67 (3.22)	-0.37 (-1.68, 0.94)	0.58	-0.72 (-1.97, 0.54)	0.26	0.22 (-0.12, 0.57)
**PHQ-9 score ≥ 5 (n [%])**	28 (43.8%)	36 (54.6%)	1.08 (0.40, 2.92)	0.89	0.65 (0.11, 3.75)	0.63	-
**Caregiver caring burden**							
**IEQ urging domain (mean [SD])**	12.14 (5.92)	14.70 (6.17)	2.31 (-0.32, 4.94)	0.085	0.78 (-1.42, 2.94)	0.49	0.13 (-0.22, 0.47)
**IEQ supervision domain (mean [SD])**	6.22 (4.92)	7.52 (5.20)	1.21 (-0.88, 3.29)	0.26	0.36 (-1.66, 2.38)	0.73	0.07 (-0.27, 0.41)
**IEQ tension domain (mean [SD])**	6.50 (5.05)	6.11 (5.67)	-1.36 (-3.04, 0.32)	0.11	-1.90 (-3.68, -0.12)	0.036	0.35 (0.003, 0.70)
**IEQ worrying domain (mean [SD])**	10.22 (6.42)	10 (6.65)	-0.75 (-3.08, 1.57)	0.53	-2.38 (-4.60, -0.16)	0.035	0.36 (0.01, 0.71)
**Reduced work due to caring (n [%])**	18 (28.1%)	22 (33.3%)	1.40 (0.60, 3.25)[c]	0.43	1.09 (0.41, 2.90)[c]	0.86	-

[a] Adjusted for sub-district (cluster) as random effect and health centre and baseline score of outcome as fixed effects
[b] Adjusted for sub-district (cluster) as a random effect and health centre, baseline score of outcome, baseline disability (proxy-reported total WHODAS), sex, baseline socio-economic status, baseline illness course, illness duration, baseline employment status, baseline caregiver employment status, baseline social support, baseline marital status, baseline treatment engagement, baseline discrimination and baseline caregiver depression (PHQ-9) as fixed effects. Illness course, illness duration and social support reduced to two categories to avoid problems with data sparsity.
[c] n=125 in minimally adjusted and fully adjusted models due to data sparsity in health centre categories.

Table 12 Secondary outcomes in persons with schizophrenia at 6 months (+/- 6 weeks)

Outcome	Facility-based care group (n=34)	CBR plus facility-based care group (n=34)	Minimally adjusted mean difference or odds ratio (95% CI)[a]	P value	Fully adjusted mean difference or odds ratio (95% CI)[b]	p value	Effect size (95% CI)
**Functioning**							
**Proxy-reported WHODAS total score (mean [SD])**	41.5 (26.6)	39.1 (23.9)	-1.82 (-15.76, 12.11)	0.80	1.97 (-12.04, 15.98)	0.78	0.08 (-0.40, 0.55)
**Proxy-reported WHODAS domain scores (mean [SD])**							
Cognition	46.6 (34.0)	44.6 (31.2)	-2.45 (-22.72, 17.82)	0.81	4.69 (-14.27, 23.64)	0.63	0.15 (-0.33, 0.62)
Mobility	20.4 (27.4)	19.7 (25.1)	-2.29 (-19.30, 14.71)	0.79	1.90 (-12.99, 16.78)	0.80	0.07 (-0.40, 0.55)
Self care	28.5 (27.2)	28.5 (28.2)	0.30 (-19.57, 20.18)	0.98	5.89 (-15.31, 27.10)	0.59	0.22 (-0.26, 0.69)
Getting along	37.8 (34.1)	39.7 (31.9)	3.81 (-16.47, 24.09)	0.71	5.58 (-11.15, 22.30)	0.51	0.17 (-0.31, 0.65)
Life activities: household	64.4 (34.8)	56.2 (34.1)	-4.58 (-21.03, 11.87)	0.59	-0.83 (-16.61, 14.95)	0.92	0.02 (-0.45, 0.50)
Life activities: work	57.8 (35.2)	53.2 (33.9)	-2.55 (-19.30, 14.20)	0.77	3.44 (-12.39, 19.28)	0.67	0.10 (-0.38, 0.58)

Participation	36.5 (26.0)	37.6 (22.2)	0.14 (-12.46, 12.75)	0.98	4.04 (-10.15, 18.22)	0.58	0.17 (-0.31, 0.65)
**Proxy-reported number of days unable work last month (Median [IQR])**	10 (0, 15)	4 (0, 15)	-2.64 (-9.10, 3.82)	0.42	-0.96 (-6.89, 4.95)	0.75	0.09 (-0.39, 0.56)
**Symptom severity**							
**At least mildly ill (CGI score ≥3) (n [%])**	28 (82.4%)	25 (73.5%)	0.53 (0.11, 2.50)[c]	0.42	0.49 (0.07, 3.24)[c]	0.46	-

[a] Adjusted for sub-district (cluster) as random effect and health centre and baseline score of outcome as fixed effects
[b] Adjusted for sub-district (cluster) as a random effect and health centre, baseline score of outcome, baseline disability (proxy-reported total WHODAS), sex, baseline socio-economic status, baseline employment status, baseline caregiver employment status, baseline social support, and baseline caregiver depression (PHQ-9) as fixed effects. Social support reduced to two categories to avoid problems with data sparsity.
[c] n=61 in minimally adjusted and fully adjusted models due to data sparsity in health centre categories.

Table 13 Primary and secondary outcomes in persons with schizophrenia at 12 months (any date)

Outcome	Facility-based care group (n=79)	CBR plus facility-based care group (n=74)	Minimally adjusted mean difference or odds ratio (95% CI)[a]	P value	Fully adjusted mean difference or odds ratio (95% CI)[b]	p value	Effect size (95% CI)
**Primary outcome**							
**Proxy-reported WHODAS-36 total score (mean [SD])**	46.2 (22.9)	40.1 (22.8)	-5.95 (-13.68, 1.79)	0.13	-7.35 (-15.01, 0.31)	0.060	0.32 (-0.003, 0.64)
**Secondary outcomes**							
**Functioning**							
**Proxy-reported WHODAS-36 domain scores (mean [SD])**							
Cognition	54.4 (29.6)	46.6 (30.4)	-6.63 (-15.34, 2.08)	0.14	-9.68 (-19.17, -0.19)	0.046	0.32 (-0.002, 0.64)
Mobility	21.3 (24.3)	19.2 (21.3)	-1.99 (-11.51, 7.52)	0.68	-1.87 (-12.33, 8.58)	0.73	0.08 (-0.24, 0.40)
Self care	32.7 (28.5)	30.5 (24.3)	-2.54 (-13.19, 8.11)	0.64	-3.03 (-12.29, 6.23)	0.50	0.11 (-0.20, 0.43)
Getting along	49.2 (30.8)	38.3 (31.1)	-10.88 (-21.37, -0.39)	0.042	-12.26 (-25.55, -0.02)	0.050	0.38 (0.06, 0.70)
Life activities: household	66.7 (33.0)	60.3 (35.3)	-6.47 (-17.37, 4.43)	0.24	-7.49 (-18.76, 3.77)	0.19	0.22 (-0.10, 0.54)
Life activities: work	6.9 (31.7)	55.6 (33.0)	-6.58 (-16.79, 3.62)	0.21	-8.84 (-19.16, 1.94)	0.11	0.27 (-0.05, 0.59)
Participation	41.8 (22.8)	36.1 (24.6)	-6.77 (-14.43, 0.90)	0.083	-8.07 (-15.98, -0.15)	0.046	0.33 (0.01, 0.65)
**Proxy-rated number of days unable work last month (Median [IQR])**	8 (2, 20)	7 (3, 15)	-2.45 (-6.01, 1.12)	0.18	-2.69 (-6.64, 1.25)	0.18	0.26 (-0.06, 0.58)
**Self-rated WHODAS total score (mean [SD]) (n=120)**	34.7 (23.7)	29.8 (19.6)	-4.05 (-11.90, 3.80)	0.31	-4.60 (-13.39, 4.19)	0.31	0.21 (-0.15, 0.57)
**Self-rated WHODAS domain scores (mean [SD]) (n=120)**							
Cognition	35.8 (29.3)	32.7 (26.7)	-0.50 (-11.02, 10.02)	0.93	-2.78 (-14.51, 8.95)	0.64	0.10 (-0.26, 0.46)

Mobility	15.9 (21.9)	17.9 (21.4)	0.34 (-9.05, 9.74)	0.94	2.69 (-7.70, 13.08)	0.61	0.12 (-0.23, 0.48)
Self-care	21.7 (23.7)	19.8 (18.4)	-1.50 (-10.48, 7.47)	0.74	-0.38 (-9.62, 8.86)	0.94	0.02 (-0.34, 0.38)
Getting along	34.4 (31.3)	24.9 (24.3)	-6.87 (-17.19, 3.45)	0.19	-7.55 (-19.03, 3.92)	0.20	0.26 (-0.10, 0.62)
Life activities: household	56.2 (35.7)	47.5 (34.1)	-7.46 (-19.66, 4.73)	0.23	-11.64 (-25.29, 2.00)	0.094	0.33 (-0.03, 0.69)
Life activities: work	50.8 (32.8)	43.0 (32.7)	-7.17 (-18.47, 4.13)	0.21	-9.21 (-21.88, 3.47)	0.15	0.28 (-0.08, 0.64)
Participation	33.4 (23.7)	27.0 (21.0)	-6.84 (-14.99, 1.32)	0.10	-6.04 (-15.90, 3.82)	0.23	0.27 (-0.09, 0.63)
**Self-rated number of days unable work last month (Median [IQR])**	4 (0,10)	5 (0,10)	-1.61 (-4.50, 1.28)	0.28	-1.75 (-5.05, 1.54)	0.30	0.23 (-0.13, 0.59)
**Proxy-rated BFS (mean [SD])**	98.87 (38.41)	87.69 (36.08)	-7.94 (-20.70, 4.82)	0.22	-6.59 (-18.11, 4.94)	0.26	0.17 (-0.14, 0.49)
**Symptom severity**							
**BPRS-E score (mean [SD]) (n=136)**	45.22 (13.83)	41.57 (15.47)	-4.10 (-9.26, 1.06)	0.12	-4.00 (-9.56, 1.56)	0.16	0.27 (-0.07, 0.61)
**At least mildly ill (CGI score ≥3) (n [%])**	64 (81%)	48 (64.9%)	0.45 (0.20, 1.01)	0.052	0.44 (0.16, 1.22)[c]	0.12	-
**Relapse**							
**Relapsed (n [%]) (n=143)**	16 (21.3%)	18 (26.5%)	1.08 (0.39, 2.98)[d]	0.88	1.49 (0.52, 4.26)[d]	0.46	-
**Health service use**							
**Non-adherent (takes medication sometimes/ occasionally/ never) (n [%])**	45 (57.0%)	22 (29.7%)	0.18 (0.06, 0.50)	0.001	0.16 (0.06, 0.44)	<0.001	-
**Any non- adherent behaviour (n [%])**	48 (60.8%)	35 (47.3%)	0.48 (0.22, 1.01)	0.054	0.56 (0.22, 1.40)	0.21	-
**No attendance at health facility for mental health last 3 months (n [%])**	38 (48.1%)	15 (20.1%)	0.23 (0.10, 0.51)	<0.001	0.15 (0.05, 0.43)	<0.001	-
**Physical restraint**							
**Restrained last 6 months (n [%])**	6 (7.6%)	5 (6.8%)	0.83 (0.18, 3.85)[e]	0.81	1.33 (0.13, 13.18) [e]	0.80	-
**Discrimination**							
**Any experience of discrimination last 6 months (n [%])**	42 (53.2%)	47 (63.5%)	1.86 (0.89, 3.89)	0.098	2.39 (0.92, 6.26)	0.075	-
**Economic activity**							
**Unemployed (n [%])**	50 (63.3%)	51 (68.9%)	1.57 (0.63, 3.92)	0.33	3.86 (0.97, 15.32)	0.055	-

[a] Adjusted for sub-district (cluster) as random effect and health centre and baseline score of outcome as fixed effects

[b]Adjusted for sub-district (cluster) as a random effect and health centre, baseline score of outcome, baseline disability (proxy-reported total WHODAS), sex, baseline socio-economic status, baseline illness course, illness duration, baseline employment status, baseline caregiver employment status, baseline social support, baseline caregiver depression (PHQ-9), residence, baseline caregiver burden, baseline alcohol use disorder and age as fixed effects. Illness course, illness duration and social support reduced to two categories to avoid problems with data sparsity.

[c] Fully adjusted model excludes baseline residence due to data sparsity.

[d] Fully adjusted model excludes baseline residence and socio-economic status due to data sparsity. n=138 in minimally adjusted and fully adjusted models due to data sparsity in health centre categories.

[e] Fully adjusted model excludes baseline residence and socio-economic status due to data sparsity. n=124 in minimally adjusted and fully adjusted models due to data sparsity in health centre categories.

Table 14 Secondary outcomes in caregivers at 12 months (any date)

Outcome	Facility-based care group (n=79)	CBR plus facility-based care group (n=74)	Minimally adjusted mean difference or odds ratio (95% CI)[a]	P value	Fully adjusted mean difference or odds ratio (95% CI)[b]	p value	Effect size (95% CI)
**Caregiver depression**							
**PHQ-9 score (mean [SD])**	4.65 (3.17)	4.51 (3.15)	-0.63 (-1.89, 0.63)	0.33	-0.55 (-1.72, 0.62)	0.36	0.17 (-0.14, 0.49)
**PHQ-9 score ≥ 5 (n [%])**	37 (46.8%)	38 (51.4%)	0.76 (0.29, 1.98)	0.57	0.77 (0.24, 2.48)	0.66	-
**Caregiver caring burden**							
**IEQ urging domain (mean [SD])**	11.90 (6.08)	13.93 (6.60)	2.37 (-0.19, 4.92)	0.069	1.68 (-0.62, 3.98)	0.15	0.26 (-0.05, 0.58)
**IEQ supervision domain (mean [SD])**	6.28 (5.12)	7.09 (5.36)	0.95 (-0.92, 2.82)	0.32	0.50 (-1.51, 2.51)	0.62	0.10 (-0.22, 0.41)
**IEQ tension domain (mean [SD])**	6.76 (5.18)	5.68 (5.53)	-1.59 (-3.17, -0.01)	0.048	-1.73 (-3.52, 0.07)	0.060	0.32 (-0.001, 0.64)
**IEQ worrying domain (mean [SD])**	10.32 (6.16)	9.38 (6.60)	-1.26 (-3.62, 1.10)	0.29	-2.10 (-4.19, -0.01)	0.049	0.32 (0.006, 0.64)
**Reduced work due to caring (n [%])**	26 (32.9%)	24 (32.4%)	0.94 (0.40, 2.19)	0.88	0.83 (0.31, 2.21)	0.72	-

[a] Adjusted for sub-district (cluster) as random effect and health centre and baseline score of outcome as fixed effects
[b] Adjusted for sub-district (cluster) as a random effect and health centre, baseline score of outcome, baseline disability (proxy-reported total WHODAS), sex, baseline socio-economic status, baseline illness course, illness duration, baseline employment status, baseline caregiver employment status, baseline social support, baseline caregiver depression (PHQ-9), residence, baseline caregiver burden, baseline alcohol use disorder and age as fixed effects. Illness course, illness duration and social support reduced to two categories to avoid problems with data sparsity.

Table 15 Secondary outcomes in persons with schizophrenia at 6 months (any date)

Outcome	Facility-based care group (n=81)	CBR plus facility-based care group (n=70)	Minimally adjusted mean difference or odds ratio (95% CI)[a]	P value	Fully adjusted mean difference or odds ratio (95% CI)[b]	p value	Effect size (95% CI)
**Functioning**							
**Proxy-reported WHODAS total score (mean [SD])**	45.5 (26.1)	43.6 (25.3)	-0.12 (-8.72, 8.48)	0.98	-1.92 (-11.19, 7.35)	0.69	0.08 (-0.25, 0.40)
**Proxy-reported WHODAS domain scores (mean [SD])**							
Cognition	51.3 (34.2)	50.9 (32.6)	2.84 (-9.49, 15.17)	0.65	2.33 (-10.61, 15.27)	0.72	0.07 (-0.26, 0.40)
Mobility	22.7 (24.6)	21.3 (24.3)	-0.90 (-8.96, 7.16)	0.83	-1.04 (-9.19, 7.11)	0.80	0.04 (-0.28, 0.37)
Self care	34.0 (29.2)	31.7 (28.9)	-2.25 (-12.41, 7.92)	0.67	-4.34 (-13.91, 5.22)	0.37	0.15 (-0.17, 0.48)
Getting along	45.5 (33.6)	45.6 (34.4)	2.76 (-8.73, 14.25)	0.64	1.02 (-10.87, 12.92)	0.87	0.03 (-0.30, 0.36)
Life activities: household	64.7 (35.6)	62.6 (33.9)	0.03 (-10.71, 10.77)	0.99	-2.63 (-14.47, 9.21)	0.66	0.08 (-0.25, 0.40)

Life activities: work	59.9 (34.7)	58.9 (33.7)	2.54 (-8.64, 13.72)	0.66	1.24 (-10.23, 12.71)	0.83	0.04 (-0.29, 0.36)
Participation	41.6 (26.2)	42.1 (23.8)	1.53 (6.73, 7.80)	0.72	-0.69 (-9.46, 8.09)	0.88	0.03 (-0.30, 0.35)
**Proxy-reported number of days unable work last month (Median [IQR])**	9 (2, 20)	6.5 (2, 21)	0.08 (-4.10, 4.26)	0.97	0.06 (-4.25, 4.37)	0.98	0.01 (0.32, 0.33)
**Symptom severity**							
**At least mildly ill (CGI score ≥3) (n [%]) (n=151)**	70 (86.4%)	58 (82.9%)	0.933 (0.33, 2.66)	0.88	0.77 (0.23, 2.58)[c]	0.68	-

[a] Adjusted for sub-district (cluster) as random effect and health centre and baseline score of outcome as fixed effects
[b] Adjusted for sub-district (cluster) as a random effect and health centre, baseline score of outcome, baseline disability (proxy-reported total WHODAS), sex, baseline socio-economic status, illness duration, baseline employment status, baseline caregiver employment status, baseline social support, baseline caregiver depression (PHQ-9), treatment engagement and symptom severity as fixed effects. Illness course, illness duration and social support reduced to two categories to avoid problems with data sparsity.
[c] Baseline treatment engagement not included in fully adjusted model. due to data sparsity. n=139 in minimally adjusted and fully adjusted models due to data sparsity in health centre categories.

Table 16 Secondary outcomes in caregivers at 12 months (+/- 10 weeks) including only those who do not differ from baseline

Outcome	Facility-based care group (n=62)	CBR plus facility-based care group (n=62)	Minimally adjusted mean difference or odds ratio (95% CI)[a]	P value	Fully adjusted mean difference or odds ratio (95% CI)[b]	p value	Effect size (95% CI)
**Caregiver depression**							
**PHQ-9 score (mean [SD])**	4.90 (3.31)	4.83 (3.20)	-1.14 (-2.68, 0.40)	0.15	-0.90 (-2.21, 0.41)	0.18	0.28 (-0.08, 0.63)
**PHQ-9 score ≥ 5 (n [%])**	29 (46.8%)	34 (54.8%)	0.73 (0.23, 2.30)	0.59	0.71 (0.12, 4.31)	0.71	-
**Caregiver caring burden**							
**IEQ urging domain (mean [SD])**	12.18 (6.37)	14.61 (5.98)	2.09 (-0.58, 4.77)	0.13	1.06 (-1.46, 3.58)	0.41	0.17 (-0.52, 0.18)
**IEQ supervision domain (mean [SD])**	6.52 (5.30)	7.77 (5.02)	1.07 (-0.91, 3.05)	0.29	0.31 (-1.88, 2.57)	0.76	0.07 (-0.29, 0.42)
**IEQ tension domain (mean [SD])**	6.76 (5.09)	6.31 (5.74)	-1.24 (-2.97, 0.49)	0.16	-1.52 (-3.46, 0.42)	0.13	0.28 (-0.08, 0.63)
**IEQ worrying domain (mean [SD])**	10.27 (6.36)	10.18 (6.56)	-0.84 (-3.16, 1.48)	0.48	-2.00 (-4.32, -0.32)	0.091	0.31 (-0.05, 0.66)
**Reduced work due to caring (n [%])**	19 (30.7%)	23 (37.1%)	1.29 (0.37, 4.53)	0.69	1.19 (0.28, 5.09)	0.81	-

[a] Adjusted for sub-district (cluster) as random effect and health centre and baseline score of outcome as fixed effects
[b] Adjusted for sub-district (cluster) as a random effect and health centre, baseline score of outcome, baseline disability (proxy-reported total WHODAS), sex, age, residence, baseline socio-economic status, baseline illness course, baseline caregiver burden (IEQ), illness duration, baseline employment status, baseline caregiver employment status, baseline social support, and baseline caregiver depression (PHQ-9) as fixed effects. Illness course and social support reduced to two categories to avoid problems with data sparsity.

Table 17 Primary and secondary outcomes in persons with schizophrenia at 12 months (+/-10 weeks) with multiple imputation

Outcome	Minimally adjusted mean difference or odds ratio (95% CI)[a]	P value	Fully adjusted mean difference or odds ratio (95% CI)[b]	P value
**Primary outcome**				
**Proxy-reported WHODAS-36 total score (mean [SD])**	-5.97 (-13.83, 1.89)	0.14	-6.98 (-14.32, 0.36)	0.062
**Secondary outcomes**				
**Functioning**				
**Proxy-reported WHODAS-36 domain scores (mean [SD])**				
Cognition	-5.93 (-14.89, 3.02)	0.19	-8.24 (-17.67, 1.20)	0.087
Mobility	-2.86 (-12.41, 6.70)	0.56	-2.45 (-12.61, 7.71)	0.64
Self care	-2.98 (-14.03, 8.07)	0.60	-2.70 (-12.88, 7.48)	0.60
Getting along	-11.94 (-22.27, -1.61)	0.024	-12.58 (-23.06, -2.11)	0.019
Life activities: household	-5.37 (-16.46, 5.71)	0.34	-6.03 (17.04, 4.97)	0.28
Life activities: work	-5.75 (-16.18, 4.68)	0.28	-7.92 (-18.41, 2.57)	0.14
Participation	-6.50 (-14.4, 1.40)	0.11	-7.21 (-15.10, 0.67)	0.073
**Proxy-reported number of days unable work last month (Median [IQR])**	-2.92 (-6.66, 0.83)	0.13	-2.56 (-6.34, 1.21)	0.18
**Self-reported WHODAS total score (mean [SD])**	-2.93 (-12.11, 6.24)	0.53	-2.57 (-12.19, 7.06)	0.60
**Self-rated WHODAS domain scores (mean [SD])**				
Cognition	2.01 (-10.35, 14.36)	0.75	0.77 (-12.03, 13.56)	0.91
Mobility	2.07 (-6.77, 10.90)	0.65	3.29 (-5.81, 12.38)	0.48
Self-care	1.79 (-11.00, 7.42)	0.70	0.79 (-9.01, 10.60)	0.87
Getting along	-6.42 (-18.33, 5.49)	0.29	-5.51 (-17.98, 6.95)	0.39
Life activities: household	-6.98 (-20.96, 7.00)	0.33	-8.33 (-22.94, 6.28)	0.26
Life activities: work	-7.11 (-20.09, 5.87)	0.28	-6.91 (-20.44, 6.61)	0.32
Participation	-5.03 (-14.39, 4.34)	0.29	-4.06 (-14.10, 5.97)	0.43
**Self-rated number of days unable work last month (Median [IQR])**	-1.58 (-4.67, 1.51)	0.32	-1.45 (-4.71, 1.80)	0.38
**Proxy-rated BFS (mean [SD])**	-7.71 (-20.33, 4.92)	0.23	-7.00 (-18.33, 4.33)	0.23
**Symptom severity**				
**BPRS-E score (mean [SD])[c]**	4.43 (-9.77, 0.92)	0.11	-3.61 (-9.34, 2.13)	0.22
**At least mildly ill (CGI score ≥3) (n [%])[c]**	OR 0.35 (0.15, 0.82)	0.015	OR 0.28 (0.09, 0.85)	0.024
**Relapse**				
**Relapsed (n [%])[d]**	OR 1.11 (0.44, 2.79)	0.82	1.42 (0.47, 4.27)	0.53
**Health service use**				
**Non-adherent (takes medication sometimes/ occasionally/ never) (n [%])**	OR 0.17 (0.07, 0.45)	<0.001	0.20 (0.07, 0.56)	0.0020
**Any non- adherent behaviour (n [%])**	OR 0.51 (0.24, 1.07)	0.073	0.60 (0.25, 1.44)	0.25
**No attendance at health facility for**	OR 0.22 (0.10, 0.51)	<0.001	OR 0.23 (0.09, 0.61)	0.0030

**mental health last 3 months (n [%])**				
**Physical restraint**				
**Restrained last 6 months (n [%])[d]**	OR 1.10 (0.24, 5.06)	0.90	OR 1.52 (0.22, 10.58)	0.67
**Discrimination**				
**Any experience of discrimination last 6 months (n [%])**	OR 1.99 (0.94, 4.24)	0.07	OR 2.26 (0.89, 5.76)	0.086
**Economic activity**				
**Unemployed (n [%])**	OR 1.64 (0.66, 4.06)	0.28	OR 3.34 (1.00, 11.21)	0.051

[a] Adjusted for sub-district (cluster) as random effect and health centre and baseline score of outcome as fixed effects
[b] Unless otherwise stated, adjusted for sub-district (cluster) as a random effect and health centre, baseline score of outcome, baseline disability (proxy-reported total WHODAS), sex, age, residence, baseline socio-economic status, baseline illness course, baseline caregiver burden (IEQ), illness duration, baseline employment status, baseline caregiver employment status, baseline social support, and baseline caregiver depression (PHQ-9) as fixed effects. Illness course and social support reduced to two categories to avoid problems with data sparsity.
[c] For BPRSE and CGI (clinician administered interview) one participant differs to all other outcomes (lay data collector interview).
[d] Health centre categories combined to avoid problems with data sparsity

Table 18 Secondary outcomes in caregivers at 12 months (+/-10 weeks) with multiple imputation

Outcome	Minimally adjusted mean difference or odds ratio (95% CI)[a]	P value	Fully adjusted mean difference or odds ratio (95% CI)[b]	p value
**Caregiver depression**				
**PHQ-9 score (mean [SD])**	-0.50 (-1.81, 0.80)	0.45	-0.28 (-1.45, 0.90)	0.65
**PHQ-9 score ≥ 5 (n [%])**	OR 0.95 (0.38, 2.41)	0.92	OR 0.93 (0.32, 2.72)	0.89
**Caregiver caring burden**				
**IEQ urging domain (mean [SD])**	2.18 (-0.31, 4.67)	0.087	1.55 (-0.84, 3.93)	0.20
**IEQ supervision domain (mean [SD])**	0.98 (-1.00, 2.96)	0.33	0.87 (-1.29, 3.03)	0.43
**IEQ tension domain (mean [SD])**	-1.64 (-3.31, 0.04)	0.055	-1.61 (-3.46, 0.25)	0.090
**IEQ worrying domain (mean [SD])**	-1.24 (-3.73, 1.25)	0.33	-2.13 (-4.25, -0.01)	0.050
**Reduced work due to caring (n [%])[c]**	OR 0.94 (0.41,2.15)	0.89	OR 1.01 (0.39, 2.65)	0.98

[a] Adjusted for sub-district (cluster) as random effect and health centre and baseline score of outcome as fixed effects
[b] Adjusted for sub-district (cluster) as a random effect and health centre, baseline score of outcome, baseline disability (proxy-reported total WHODAS), sex, age, residence, baseline socio-economic status, baseline illness course, baseline caregiver burden (IEQ), illness duration, baseline employment status, baseline caregiver employment status, baseline social support, and baseline caregiver depression (PHQ-9) as fixed effects. Illness course and social support reduced to two categories to avoid problems with data sparsity.
[c] Health centre categories combined to avoid problems with data sparsity

Table 19 Secondary outcomes in persons with schizophrenia at 6 months (+/- 10 weeks) with multiple imputation

Outcome	Minimally adjusted mean difference or odds ratio (95% CI)[a]	P value	Fully adjusted mean difference or odds ratio (95% CI)[b]	p value
**Functioning**				
**Proxy-reported WHODAS total score (mean [SD])**	-1.07 (-10.96, 8.83)	0.83	-1.99 (-12.36, 8.38)	0.71
**Proxy-reported WHODAS domain scores (mean [SD])**				
Cognition	0.70 (-12.79, 14.20)	0.92	1.48 (-12.54, 15.49)	0.84
Mobility	-1.12 (-11.6, 9.40)	0.83	-1.82 (-12.94, 9.31)	0.75
Self care	-2.64 (-13.95, 8.67)	0.65	-3.80 (-15.43, 7.82)	0.52
Getting along	5.10 (-8.56, 18.76)	0.46	3.71 (-10.59, 18.02)	0.61
Life activities: household	-2.75 (-15.83, 10.32)	0.68	-3.97 (-17.61, 9.67)	0.57
Life activities: work	-0.96 (-14.15, 12.23)	0.89	-0.80 (-14.12, 12.53)	0.91
Participation	-2.56 (-12.63, 7.51)	0.62	-3.50 (-14.00, 6.99)	0.51

**Proxy-reported number of days unable work last month (Median [IQR])**	-1.13 (-5.32, 3.06)	0.60	-1.34 (-5.86, 3.18)	0.56
**Symptom severity**				
**At least mildly ill (CGI score ≥3) (n [%])[c]**	OR 0.79 (0.25, 2.48)	0.69	OR 0.71 (0.17, 3.03)	0.64

[a] Adjusted for sub-district (cluster) as random effect and health centre and baseline score of outcome as fixed effects
[b] Adjusted for sub-district (cluster) as a random effect and health centre, baseline score of outcome, baseline disability (proxy-reported total WHODAS), sex, baseline socio-economic status, baseline employment status, travel time to health facility, baseline caregiver employment status, baseline social support, and baseline caregiver depression (PHQ-9) as fixed effects. Social support reduced to two categories to avoid problems with data sparsity.
[c] Health centre categories combined to avoid problems with data sparsity

References

1. Habtamu K, Alem A, Medhin G, Fekadu A, Dewey M, Prince M, et al. Validation of the World Health Organization Disability Assessment Schedule in people with severe mental disorders in rural Ethiopia. Health and quality of life outcomes. 2017;15(1):64.
2. Habtamu K, Alem A, Medhin G, Fekadu A, Prince M, Hanlon C. Development and validation of a contextual measure of functioning for people living with severe mental disorders in rural Africa. BMC Psychiatry. 2016;16(1):311.
3. Susser E, Finnerty M, Mojtabai R, Yale S, Conover S, Goetz R, et al. Reliability of the life chart schedule for assessment of the long-term course of schizophrenia. Schizophr Res. 2000;42(1):67-77.
4. Chisholm D, Sekar K, Kumar KK, Saeed K, James S, Mubbashar M, et al. Integration of mental health care into primary care. Demonstration cost-outcome study in India and Pakistan. Br J Psychiatry. 2000;176:581-8.
5. Mogga S, Prince M, Alem A, Kebede D, Stewart R, Glozier N, et al. Outcome of major depression in Ethiopia. The British Journal of Psychiatry. 2006;189(3):241-6.
6. Chatterjee S, Leese M, Koschorke M, McCrone P, Naik S, John S, et al. Collaborative community based care for people and their families living with schizophrenia in India: protocol for a randomised controlled trial. Trials. 2011;12(1):12.
7. Chatterjee S, Naik S, John S, Dabholkar H, Balaji M, Koschorke M, et al. Effectiveness of a community-based intervention for people with schizophrenia and their caregivers in India (COPSI): a randomised controlled trial. The Lancet. 2014;383(9926):1385-94.
8. Thornicroft G, Brohan E, Rose D, Sartorius N, Leese M. Global pattern of experienced and anticipated discrimination against people with schizophrenia: a cross-sectional survey. Lancet. 2009;373(9661):408-15.
9. Mortimer AM. Symptom rating scales and outcome in schizophrenia. The British Journal of Psychiatry. 2007;191(50):s7-s14.
10. Gonda T, Deane FP, Murugesan G. Predicting clinically significant change in an inpatient program for people with severe mental illness. Aust N Z J Psychiatry. 2012;46(7):651-8.
11. Burlingame GM, Seaman S, Johnson JE, Whipple J, Richardson E, Rees F, et al. Sensitivity to change of the Brief Psychiatric Rating Scale - Extended (BPRS-E): an item and subscale analysis. Psychological Services. 2006;3(2):77-87.

12. Youngmann R, Zilber N, Workneh F, Giel R. Adapting the SRQ for Ethiopian populations: a culturally-sensitive psychiatric screening instrument. Transcultural psychiatry. 2008;45(4):566-89.
13. Hanlon C, Alem A, Medhin G, Shibre T, Ejigu DA, Negussie H, et al. Task sharing for the care of severe mental disorders in a low-income country (TaSCS): study protocol for a randomised, controlled, non-inferiority trial. Trials. 2016;17(1):76.
14. Burlingame GM, Dunn TW, Chen S, Lehman A, Axman R, Earnshaw D, et al. Selection of outcome assessment instruments for inpatients with severe and persistent mental illness. Psychiatr Serv. 2005;56(4):444-51.
15. van Wijngaarden B, Schene AH, Koeter M, Vazquez-Barquero JL, Knudsen HC, Lasalvia A, et al. Caregiving in schizophrenia: development, internal consistency and reliability of the Involvement Evaluation Questionnaire--European Version. Br J Psychiatry Suppl. 2000(39):s21-7.
